# High-Yield Synthesis of Cu_29_ Nanoclusters and Their Applications in Photothermal Conversion and Catalysis

**DOI:** 10.1021/acs.inorgchem.5c01411

**Published:** 2025-08-22

**Authors:** Avirup Sardar, Yitong Wang, Abhrojyoti Mazumder, Guiying He, Christopher G. Gianopoulos, Kristin Kirschbaum, Rongchao Jin

**Affiliations:** † Department of Chemistry, 6612 Carnegie Mellon University, Pittsburgh, Pennsylvania 15213, United States; ‡ Department of Chemistry and Biochemistry, 7923 University of Toledo, Toledo, Ohio 43606, United States

## Abstract

Atomically precise copper nanoclusters (NCs) have received considerable interest in recent years. Significant progress is being made in understanding their synthesis, size/shape control, and crystallization techniques. Unlike Au and Ag nanoclusters, zerovalent Cu NCs are much more difficult to synthesize due to the low reduction potential of Cu­(II) or Cu­(I). However, Cu is unique in its catalytic reactivity and photothermal conversion. This study presents a high-yield procedure for bulk synthesis of a Cu_29_ NC (in Cu­(I) valence state) coprotected by cyclohexanethiolate (CHT) and triphenylphosphine (TPP), with its crystal structure characterized. The Cu_29_–CHT-TPP is further investigated as an effective photothermal material as well as a catalyst for the azide–alkyne click-chemistry reactions. The Cu_29_ NC possesses a good photothermal activity (e.g., a 22.4 °C increase in 450 s for 0.4 OD_488 nm_ when irradiated 488 nm laser of 1.75 W/cm^2^) and a photothermal efficiency of 33%, which rivals the best Au NCs reported. In the catalysis, it shows fast reactions and >90% yields under blue LED irradiation, along with good recyclability, when used as a homogeneous catalyst. The findings from this work may promote future explorations of Cu NCs in various applications.

## Graphical abstract

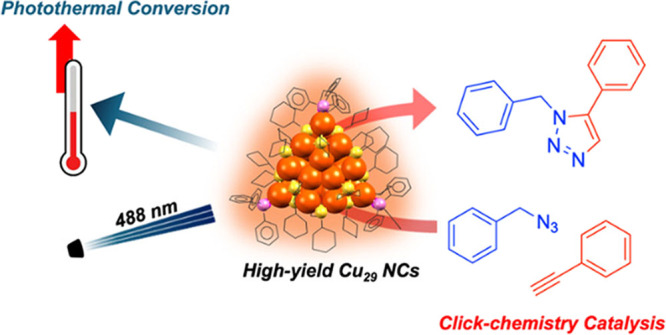

## Introduction

In recent years, atomically precise metal nanoclusters (NCs) have gained wide research interest due to their potential applications in optics, biomedicine, electrochemistry, catalysis, etc.^1–6^ Unlike their metal complex or nanoparticle counterparts, metal NCs exhibit quantized energy levels and tunable HOMO–LUMO gaps. The quantized electronic structure leads to distinct peaks in the optical absorption spectra, which can be modified by controlling the ligands or tinkering with their synthesis, thereby producing favorable structures and properties.^7^

Compared to the gold and silver nanoclusters—which have been studied extensively due to their easier synthesis and stability,^8,9^ research on Cu NCs is still limited^10^ despite Cu being in the same Group 11 as Au and Ag. This is mainly due to the low reduction potential (Cu^2+^/Cu = 0.34 V, Cu^+^/Cu = 0.52 V) of Cu in comparison to the other coinage metals. Due to this difficulty in the reduction of Cu precursors, there are only a handful of Cu(0) NCs reported to date,^11–13^ and the synthesized Cu(0) NCs are also extremely prone to oxidation. On the other hand, Cu­(I) NCs are quite common in the literature.^2^ Nevertheless, high-yield synthesis of high-purity Cu­(I) NCs is still far-fetched, and mechanistic understanding is also less. Therefore, studies on developing high-yield synthesis and purification techniques are particularly important.^10^ Cu complexes have long been used since the 1900s for various photophysical and catalytic applications, along with Cu nanoparticles in recent decades.^14–17^ Currently, Cu­(I) NCs have shown promising applications in photothermal conversion and organic catalysis.^18–22^ Their higher surface area and smaller size than those of the nanoparticle counterparts facilitate significantly better catalytic properties due to more active sites. Recently, researchers have started exploring catalysis by Cu NCs, but the versatility with various types of clusters is still under investigation.^23^ While Au NCs have been studied for photothermal conversion and near-infrared absorptivity,^24,25^ similar studies with copper are still rare.^12^

Herein, we report a high-yield (71%, Cu atom basis), high-purity strategy for the synthesis of [Cu_29_(CHT)_18_(TPP)_4_H_10_]­BF_4_ (where CHT = cyclohexanethiolate and TPP = triphenylphosphine), abbrev. Cu_29_, and demonstrate the various routes of obtaining the same product, showing the versatility of Cu_29_, which can be useful for future research on Cu NCs. We discuss the role of various reducing agents and how they affect the yield of the product and minimize the byproducts. We then describe different crystallization procedures used to obtain (i) crystalline samples, and (ii) high yield, both of which are still elusive in Cu NC research. The obtained crystals are solved by single-crystal X-ray diffraction (sc-XRD). In the investigation of properties, a comparison is drawn between the excited state relaxation properties of Cu_29_ and the previously reported Cu_28_ with the same ligands. Photothermal experiments are also performed with the Cu_29_ NC, which shows a high photothermal conversion efficiency (∼33%). Finally, we investigate Cu_29_ as an effective catalyst for [2 + 3] Huisgen 1,3-dipolar cycloaddition and azide–alkyne click-chemistry reactions, which show fast reaction rates, good recyclability, and >90% yields.

## Results and Discussion

### Synthetic Design and Crystallization

The Cu_29_ NC was synthesized by a modified procedure used by various groups^26–29^ in preparing Cu­(I) NCs (Figure 1). Briefly, Cu­(CH_3_CN)_4_·BF_4_ and triphenylphosphine (TPP) in a 1.5:1 ratio were added to a solvent mixture (6 mL) of 1:1 chloroform and acetonitrile, followed by the addition of cyclohexanethiol. The reaction mixture was then subject to slow reduction by either an ice-cold NaBH_4_ solution or *tert*-butylamine borane complex (^*t*^BuNH_2_·BH_3_) to form NCs. The as-synthesized NCs were then dissolved in suitable solvents for crystallization. Orange-red crystals were obtained (Figure S1). For all subsequent experiments, we used Cu_29_ solutions by redissolving crystals to ensure no interference from impurities.

**Figure 1:**
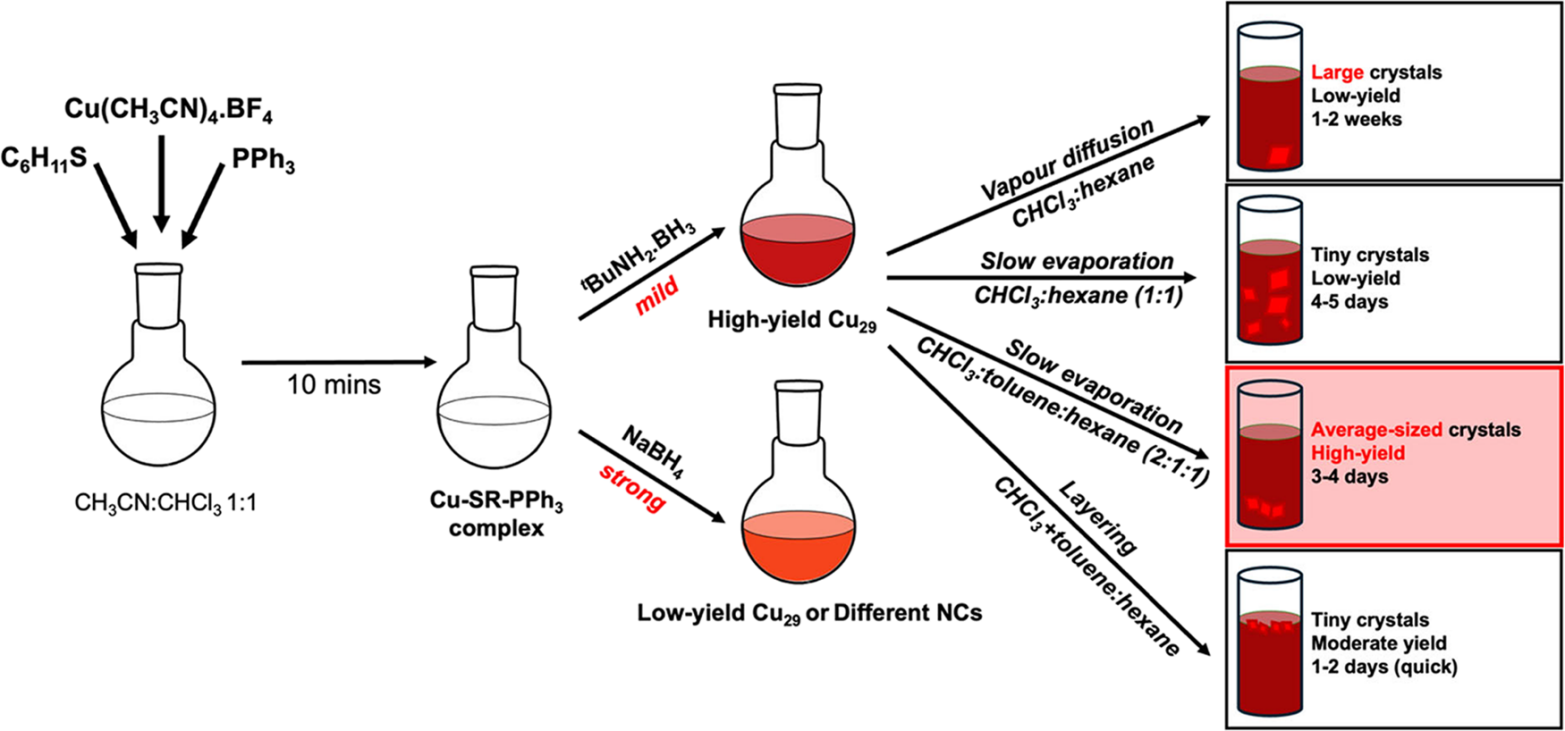
High-yield synthetic procedure and crystallization techniques used to prepare the Cu_29_–CHT-TPP.

It is common knowledge that the ratio of the precursors, solvents, and reducing agents plays an important role in the purity and yield of the Cu NCs. In our work, after trying various solvent systems (Table S1), we find that a 1:1 mixture of chloroform and acetonitrile while using ^*t*^BuNH_2_.BH_3_ (borane complex) as the reducing agent provided the highest yield of 71% (on a Cu atom basis) for Cu_29_ NCs. Negishi et al.^30^ recently reported the direct synthesis of a Cu_14_ NC using an equimolar amount of Cu-precursor and TPP while using NaBH_4_ as the reducing agent. In previous studies, using a low Cu-to-TPP ratio mediated the formation of unwanted Cu-TPP complexes (the side-products),^37^ hence, a low yield of NCs. On the other hand, high ratios did not facilitate the formation of the Cu-TPP intermediate that is crucial for the NC growth. Based on this knowledge, we change the Cu-precursor/TPP ratio to 1.5:1 while keeping other conditions, which leads to the formation of Cu_29_, instead of Cu_14_, with a yield of 30%. Further, if we change the reducing agent to a *tert*-butylamine borane complex, Cu_29_ NCs are obtained exclusively with high yield (71%). This shows that the Cu NC synthesis is extremely sensitive to the reaction conditions, and tinkering with various precursor ratios, and more importantly, the reducing agent can affect the product as well as the purity.

Purification is extremely important for studying the properties of NCs with accuracy, and crystallization is one of the most common procedures to obtain high-purity NCs. Also, high-quality crystals are beneficial for solving their structures, and such structures are the basis for understanding the structure–property relationship.^31–33^ In this work, we have tried various crystallization methods with a variety of solvents and found some systems that provide better quality crystals and high-yield crystallization. Specifically, crystallizing the crude Cu_29_ NCs by diffusing hexane into a chloroform solution of the NCs results in better quality crystals in 1–2 weeks, which were used for sc-XRD. However, slow evaporation of the NCs in a 2:1:1 chloroform:toluene:hexane provides the highest yields of the Cu_29_ crystals in 3–4 days. On the other hand, layering hexane into a chloroform:toluene mixture of the NCs gives rise to crystals in just 1–2 days, although the quality is poorer. Overall, it is important to study different crystallization methods of Cu NCs to determine the most efficient crystallization methods to achieve the maximum purity for further experiments.

### Crystal Structure and Hydrido Determination

The Cu_29_ NC crystallizes in a trigonal crystal system with the *R*3̅ space group (Table S2). Single crystal X-ray diffraction analysis identified [Cu_29_(CHT)_18_(TPP)_4_] (Figure 2a, Tables S3 and S4), but electrospray ionization mass spectrometry (ESI-MS) analyses of the Cu_29_ and the deuterated-Cu_29_ (Cu_29_D) revealed that the NC bears 10 hydrido atoms (see below), so, the full formula of the NC is [Cu_29_(CHT)_18_(TPP)_4_H_10_]­BF_4_. Of note, hydrido atoms are not resolved in sc-XRD.^3,4^ Comparing with similar ones reported in the literature,^18,27,31,34^ our cationic Cu_29_ bears one BF_4_^–^ (unresolved by sc-XRD but confirmed by MS, see below) as the counterion.

**Figure 2:**
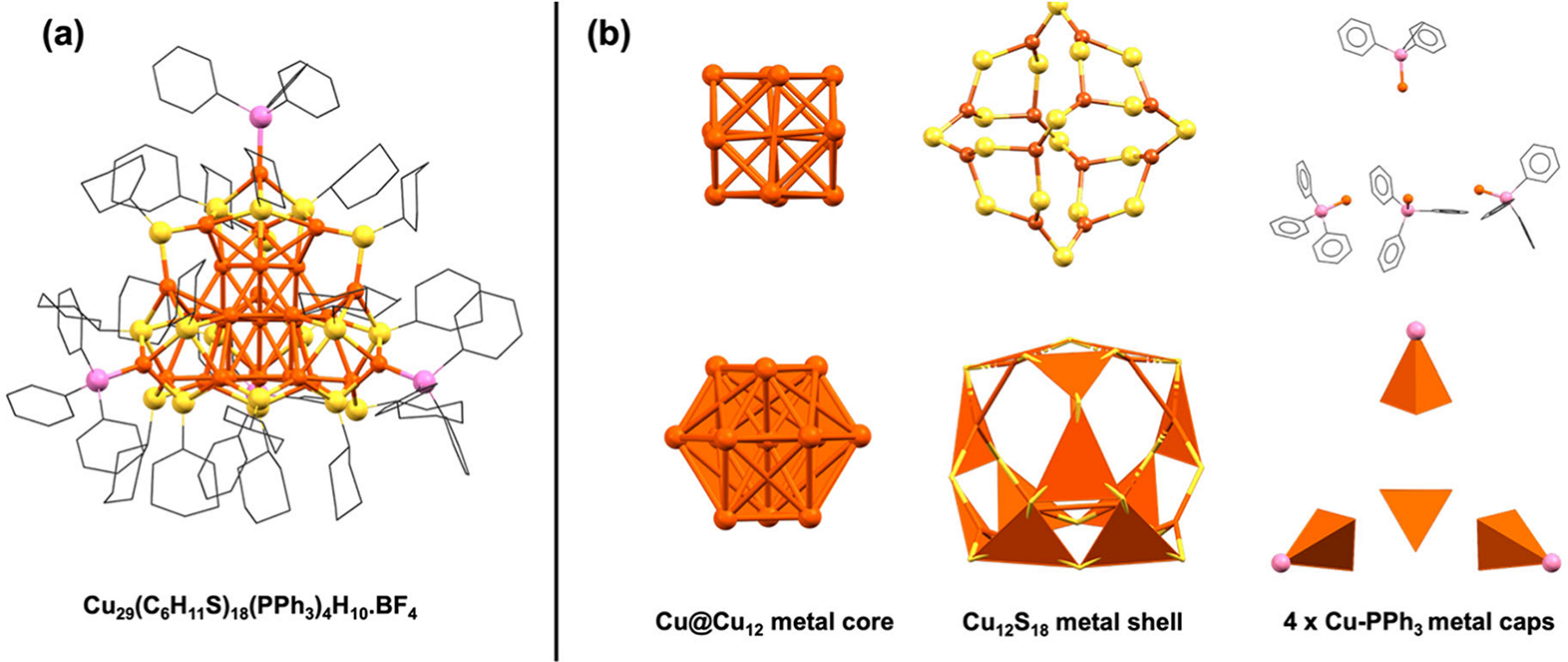
(a) Full structure of Cu_29_(CHT)_18_(TPP)_4_H_10_·BF_4_ (BF_4_^–^ and hydride atoms not resolved, H atoms omitted); (b) anatomy of the Cu_29_ structure, including the Cu@Cu_12_ core, Cu_12_S_18_ shell, and 4 Cu-PPh_3_ caps.

The structure of the NC is similar to the various Cu_29_ NCs reported in the literature,^31,34,35^ but differs from the icosahedral one.^36^ The structure can be viewed as a core–shell architecture.^1–4^ The cuboctahedral Cu@Cu_12_ forms the core, which is covered by a Cu_12_(SR)_18_ shell, and further by four Cu-PPh_3_ caps (Figure 2b). The Cu_12_(SR)_18_ shell can be divided into four distorted hexagonal units of Cu_3_S_3_ connected by 6 SR^–^ bridges (Figure S2). The 18 thiolates and 4 phosphines come from the precursors, whereas the hydrides are confirmed to come from the reducing agent, not the solvents, because when the reaction was performed in deuterated solvents, the same Cu_29_ NC with 10 hydrides (H) was obtained, but when we used the deuterated borane complex (^t^BuNH_2_·BD_3_), we observed an increase of the NC mass by ∼10 Da in ESI-MS analysis (see below). It is also worth noting that out of the Cu_29_–SR-TPP NCs in the literature,^31,34,35^ our Cu_29_ lacks the Cl^–^ ligands in the structure, whereas the reported Cu_29_–SR-TPP NCs with S-^*t*^Bu or S-Adm have 5/6 or 3 Cl^–^ ligands, respectively (Figure S3). This is most probably due to the presence of 18 CHT and 10 hydrides in our Cu_29_, which leaves no more space for the Cl^–^ ligands to bind to the Cu atoms, as in the case of other isostructural Cu_29_–CHT NCs.^34^ Unlike the other NCs where researchers have confirmed the source of the chloride to be from the solvent mixtures by changing chloroform to tetrahydrofuran (THF), we performed control experiments by changing the solvents from a chloroform:acetonitrile mixture to a 1,2-dichloroethane:acetonitrile mixture (which has more chloride source) but still obtained the same Cu_29_ NC, proving that the chlorides do not attach to our Cu_29_.

To further confirm the formula of the Cu_29_ NC obtained from the crystal structure, we performed an ESI-MS analysis. The positive mode ESI-MS spectrum shows a strong peak at 4975.56, which corresponds to [Cu_29_(CHT)_18_(TPP)_4_H_10_]^+^ (Figure 3a). The experimental isotope pattern matches the simulated pattern (Figure 3a, inset). The isotopic pattern also shows a Δ*m*/*z* = 1 spacing, which indicates *a* + 1 charge (Figure S4). We also performed the ESI-MS analysis of deuterated Cu_29_ and found a strong peak at 4985.70, corresponding to [Cu_29_(CHT)_18_(TPP)_4_D_10_]^+^. This verifies the 10 hydrides (Figure 3b). In addition, the negative mode ESI-MS (Figure S5) shows a peak at *m*/*z* 87 (*z* = −1), confirming the BF_4_^–^ counterion.

**Figure 3:**
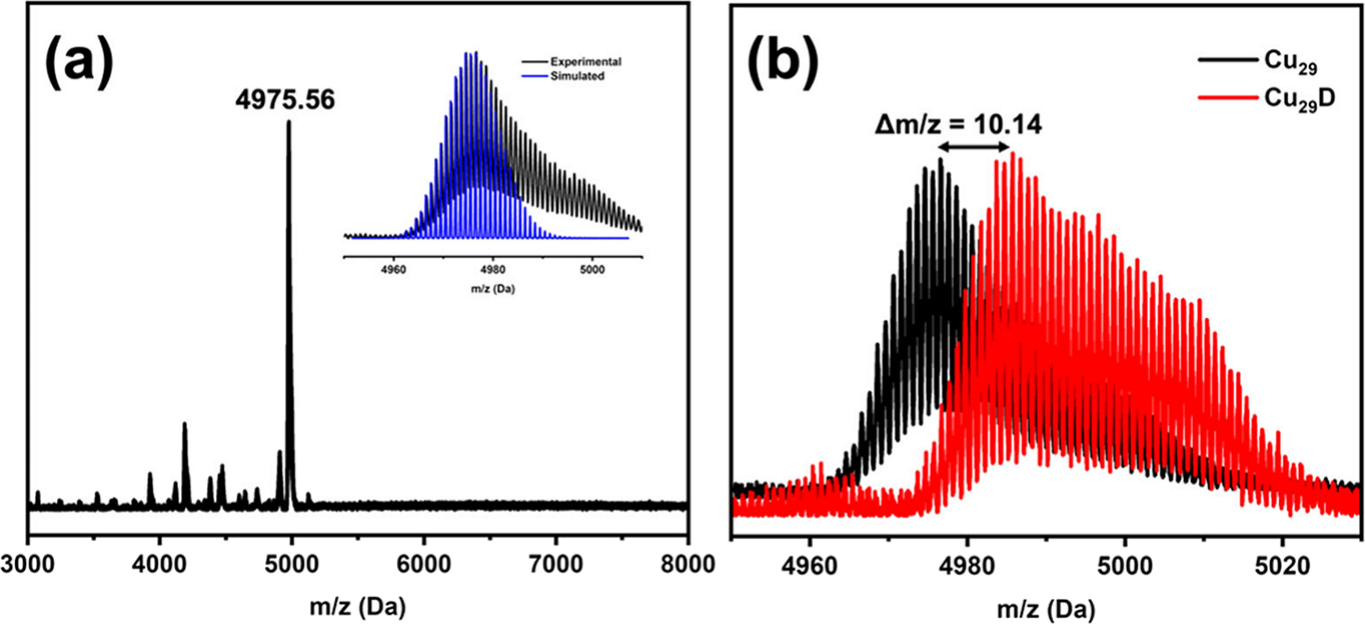
(a) ESI-MS spectrum of Cu_29_, inset: the simulated isotope pattern (blue) and the experimental pattern (black); (b) Comparison between ESI-MS of Cu_29_ and its deuterated analogue Cu_29_D.

### Optical Absorption and Excited State Dynamics

The Cu_29_ NC shows a featureless UV–vis spectral profile with a monotonic decrease with increasing wavelength (Figure 4a), which is quite common for Cu­(I)-hydride systems.^2^ The spectrum is consistent in different solvents, e.g., dichloromethane, chloroform, and tetrahydrofuran. The time-dependent UV–vis spectra show that the Cu_29_ is photostable even up to 15 days (Figure S6) when stored under mild conditions and also after performing various experiments.

**Figure 4:**
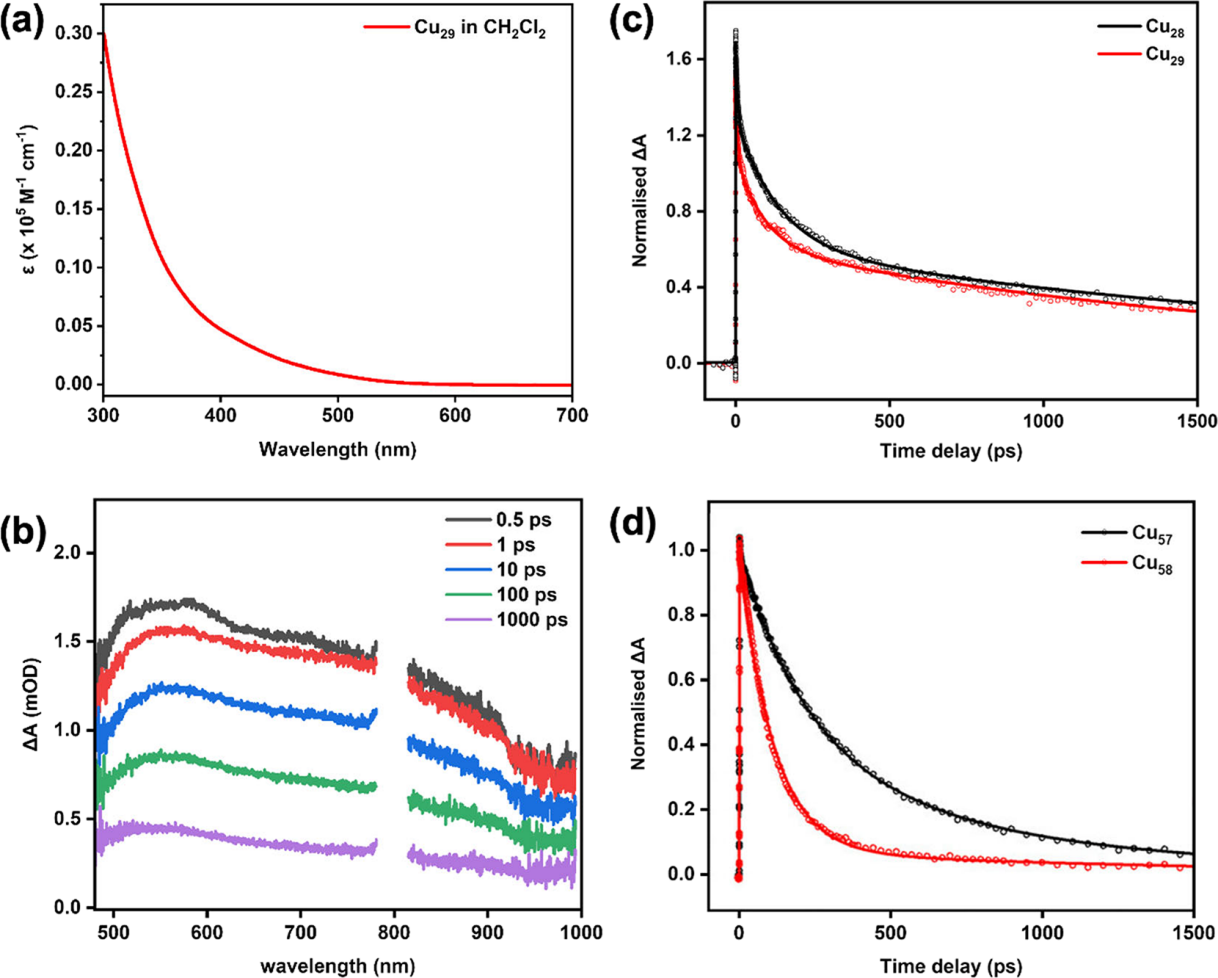
(a) UV–vis absorption spectrum of Cu_29_ in dichloromethane; (b) transient absorption spectra of Cu_29_ at different time delays, excited at 400 nm. Note: Data points around 800 nm were omitted due to instrumental artifacts; (c) Normalized fs-TA kinetic traces for Cu_29_ and Cu_28_ detected at 700 nm; (d) The reported Cu_58_ and Cu_57_ fs-TA kinetic traces detected at 640 nm. Fitting curves are solid lines, and original data points are in circles. Note: panel d is reproduced with permission from ref 37 , 2023 Wiley-VCH GmbH.

Considering the reported photoinduced click reaction of Cu NCs, we are curious about the photophysical properties of Cu_29_. To investigate the excited state dynamics, we performed femtosecond transient absorption (fs-TA) measurements since no obvious photoluminescence was observed from Cu_29_. It is worth noting that Bakr et al.^37^ previously demonstrated how defects in the Cu NC structures (Cu_58_ and the one-atom lost Cu_57_) can affect the excited-state properties. In our work, we investigated the excited state properties of our Cu_29_ and the reported Cu_28_.^34^ The fs-TA spectra of Cu_28_ and Cu_29_ in dichloromethane solutions show similar TA spectra and ultrafast dynamics (Figures 4b and S7), and both Cu_28_ and Cu_29_ solutions, as well as their thin films, exhibit a broad positive feature from 480 to 950 nm at various time delays, corresponding to photoinduced excited state absorption. The fitted time constants reveal that both NCs have a triexponential decay (Figure S8). The fast decay component (∼6 ps, amplitude ∼25%) for both NCs is attributed to the relaxation of geometry. While the subsequent excited state relaxation shows a slower decay in Cu_28_ (137 ps) than in Cu_29_ (91 ps) (Figures S8 and 4c), similar to the Cu_58_–Cu_57_ system,^37^ but the latter pair shows a more marked difference in the excited state absorption (Figure 4d). The lifetime of the final excited state is around 2.2 ns, which may be considered as the nonradiative relaxation pathway. The relatively long-lived excited states of Cu_28_ and Cu_29_ should benefit the photothermal and photoinduced reactions.

The difference between the two pairs can be explained by their electronic structures from previous calculations (Figure S9). Cu_58_ and Cu_57_ show a marked difference in their electronic transitions by comparing the hole–electron maps of their optimized structures, with Cu_58_ showing charge transfer from the outer metal shells to the inner metal shells, but Cu_57_ showing charge transfer from the vacancy site toward the surface ligands. If we compare them with the optimized structures of Cu_29_–Cu_28_ from the literature,^31,50^ we see that the frontier molecular orbitals (FMOs) have a much greater resemblance than in the case of Cu_58_–Cu_57_. Also, removing one Cu atom from the highly symmetric Cu_58_ has a larger effect on the charge transfer dynamics than removing one atom from the noncubic Cu_29_ structure.

Overall, we conclude that it is difficult to differentiate defected Cu NCs in both steady-state and excited state analyses, at least not in the Cu_29_ vs Cu_28_.

### Photothermal Conversion

Recently, materials showing photothermal conversion have garnered significant research interest due to their potential applications in heating agents, thermal imaging, and solar cells.^16,38,39^ Cu nanoparticles have been widely used as photothermal conversion agents for quite some time.^38^ Gold NPs^40^ and NCs^24,25^ have also been studied as good photothermal conversion agents in recent years. However, Cu NCs for photothermal studies are still rare.^12^ Most of the studies in recent times are on near-infrared photothermal therapy, which utilizes the deep-tissue penetration ability of near-infrared light for phototherapy. However, for surface-tissue treatments, especially for superficial skin (tissue penetration for light <440 nm: 0.3–0.5 mm, light of 440–600 nm: 1–2 mm, light >600 nm: 2–11 mm),^41,42^ blue-light therapy (400–500 nm) is much more crucial. Recent studies have shown how light of various wavelengths targets particular skin depths, implying the importance of studying photothermal therapy and photodynamic therapy of materials using different wavelengths.^43,44^ Photothermal materials coupled with lasers in the range of 450–500 nm are currently showing marked success in treating melanoma as well as other types of cancer.^45,46^ Here, we measured the photothermal conversion of Cu_29_. Figure S10 shows the setup used in our experiments.

We dissolved Cu_29_ crystals in 0.5 mL of chloroform (0.4 OD at 488 nm). We note that toluene is one of the most common media to perform photothermal conversion; however, due to the Cu_29_ solubility issue, here we use CHCl_3_ instead. A 488 nm laser was used to excite the NCs since the UV–vis profile of Cu_29_ shows absorption in the 300–500 nm region. As seen from Figure 5a,b and Table S5, the temperature rises to 43.5 °C from the starting temperature of 21.1 °C over a period of 450 s when 1.75 W cm^–2^ of 488 nm laser light irradiates the solution, i.e., an increase of 22.4 °C. After 600 s, it gradually cools down. For a blank test in chloroform, no temperature increase was seen. We calculate the photothermal conversion efficiency (η) by the following equation:^48,49^

$$\eta =\frac{hS\Delta T_{\max}}{I(1-10^{-A_{\lambda }})}$$ 1

where *h* is the heat transfer coefficient, *S* is the surface area of the vial, Δ*T*_max_ is the temperature change, *I* is the incident laser power density, and *A*_λ_ is the absorbance of the NCs at 488 nm wavelength.

**Figure 5:**
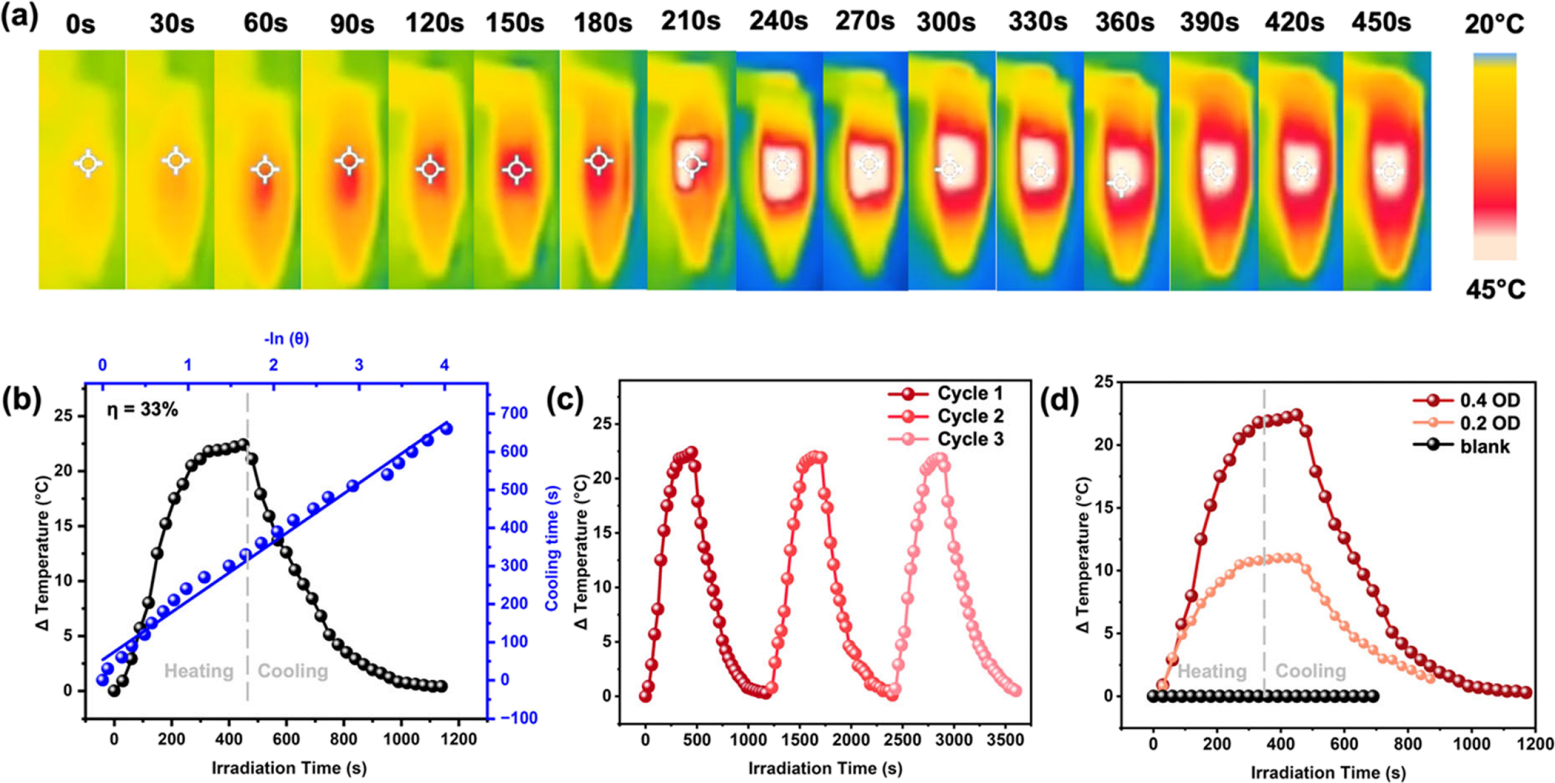
(a) Time-dependent thermal images of Cu_29_ in CHCl_3_ during the heating (taken by thermal camera) upon laser irradiation; (b) heating and cooling curves of Cu_29_ and plot of cooling time vs. the negative logarithm of the temperature driving force (θ) during the cooling stage; (c) temperature change of Cu_29_ solutions over three laser on/off cycles; (d) temperature change of solutions with concentrated (maroon, 0.4 OD at 488 nm), dilute (orange, 0.2 OD at 488 nm) Cu_29_, and blank CHCl_3_ (black). Laser irradiation: 488 nm, 1.75 W cm^–2^.

From the temperature decay curve, we calculated the sample heating time constant τ_s_ to be 154.9 s. Plugging other values into the equation determines the photothermal efficiency of Cu_29_ to be 33% (see details in the Supporting Information). This efficiency remained similar in three cycles (Figure 5c), indicating the photostability of Cu_29_. The results are thereby very promising and are also comparable to the reported photothermal efficiency of the Au_52_ and Au_60_ NCs.^24,25^ The UV–vis spectra before and after the photothermal experiments were similar (Figure S11), which again confirms the excellent photostability of Cu_29_. We also performed similar experiments with a lower concentration, 0.2 OD at 488 nm. With similar 488 nm laser irradiation time, the maximum temperature rise was ∼11 °C (Figures 5d and S12), which is almost half of that obtained at the higher concentration (0.4 OD). This is consistent with the above equation and verifies the concentration dependence in photothermal conversion by the clusters under the experimental conditions. After the laser is off, the cooling of the NC solution follows Newton’s law:

$$T(t)=T_{s}+(T_{0}-T_{s})e^{-kt}$$ 2

After the NCs cool to ambient temperatures, we find the average value of the cooling constant (*k*) value to be 0.005 s^–1^, which is similar to Au_42_ NCs discussed before. In consideration of the lower absorptivity of Cu_29_ than Au_42_, the high photothermal conversion efficiency of Cu_29_ is thus quite extraordinary, which can be attributed to the stronger electron–phonon coupling in Cu than in Au NCs.^47^

### Catalytic Reactivity in Click Chemistry Reactions

We further demonstrate the catalytic application of Cu_29_ in click chemistry, specifically the Azide–Alkyne cycloaddition reactions (AAC). Click chemistry reactions are a subject of strong interest due to their simplicity and the tendency to form a single product. Cu NPs and also copper complexes have been widely used to perform organic catalysis for a long time.^50–52^ Previously, Au NCs were investigated as catalysts for various types of organic reactions and showed a limited to good applicability.^53–55^ Currently, researchers have shown how Cu NCs can act as even better catalysts than their NP and complex counterparts due to their high surface area and more active sites.^19,56–59^ Among the click-chemistry reactions, Cu-catalyzed AAC is one of the most famous, and recent work has been awarded the Nobel Prize in 2022. These reactions are much more effective than thermal organic reactions, as one can target specific reaction sites and products with photoexcitation of the catalysts. Here, we evaluate the effectiveness of Cu_29_ in AAC catalysis reactions.

The catalytic reactions were performed in *d*-chloroform (3 mL) with 0.02 mol Cu_29_ as the catalyst under blue light irradiation for 1 h (Figure S13). Of note, we used *d*-chloroform instead of the regular chloroform so that we can easily track the progress of the reaction through NMR. The Cu_29_ absorbs in the 300–500 nm region, so the NCs can utilize energy from the light for the catalysis, as reported before.^19,31,57^ The NCs are found to be stable in blue light for a long time, and the UV–vis spectrum shows no loss. The model reaction between phenylacetylene and benzyl azide is catalyzed by Cu_29_, with a yield of 91% (Table 1). We performed the same reaction under dark conditions and obtained a yield of 35% only, and a zero yield when there was no catalyst. Control experiments are also performed with equimolar Cu-CHT complex or Cu­(CH_3_CN)·BF_4_ salt, but the reaction yields are significantly lower (52 and 62%, respectively) than the case of Cu_29_ as the catalyst. After each run, we obtained the products by column chromatography, and Cu_29_ showed no degradation of its absorption spectrum (Figure S14). The Cu_29_ catalyst showed good efficiency up to 3 reaction cycles; the yield decreased from its initial 91 to 85% for the second cycle and 73% for the third cycle. We also increased the substrate scope and performed the AAC reactions with 3-ethynyltoluene, 4-ethynyltoluene, 4-*tert*-butylphenylacetylene, and 1-octyne. The yields in these cases are 83, 81, 88, and 60%, respectively (Table 1). The NMR spectra of the products are provided in the Supporting Information (Figures S15–S19). Interestingly, while performing the catalytic experiments with Cu_28_ instead of using our NCs, we obtained similar yields under identical reaction conditions (Table S6).

**Table 1:**
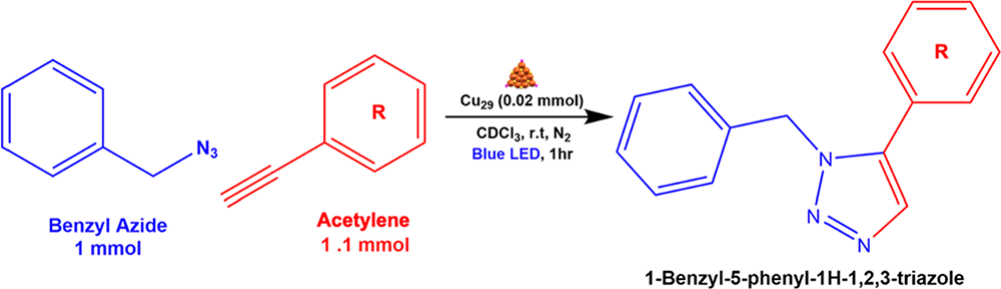
Cu_29_-Catalyzed Azaide-Alkyne Cycloaddition Reactions with Various Acetylenes

variation of conditions		
no.	conditions	yield (%)
1.	standard	91
2.	no catalyst	0
3.	dark	35
4.	Cu-CHT complex	52
5.	Cu(CH_3_CN)_4_.BF_4_ salt	62

variation of alkynes		
no.	alkyne used	yield
1.	phenylacetylene	91
2.	3-ethynyltoluene	83
3.	4-ethynyltoluene	81
4.	4-*tert*-butylphenylacetylene	88
5.	1-octyne	60

Based on previous works performed by other groups^37,59^ we ascertain that the click-chemistry reactions catalyzed by our NCs undergo a photoinduced intramolecular single-electron transfer (SET) process, followed by a radical pathway proposed before. The process involves the formation of a CuNC-alkynyl-azide complex followed by SET, N-radical attack, and back-electron donation, which forms the desired triazole product via cyclization as well as regenerates the CuNCs. Overall, the Cu_29_ NC from this work shows good activity in catalyzing the click chemistry reactions.

## Conclusions

In summary, we have synthesized a Cu_29_ nanocluster protected by cyclohexanethiolate and triphenylphosphine and investigated how tinkering with the precursors, solvent ratio, and reducing agents can lead to high-yield synthesis of copper NCs. We compared the excited state absorption of Cu_29_ with the reported Cu_28_ and drew a comparison with the Cu_58_–Cu_57_ systems to show how the defects and overall size affect the properties. Due to its absorption of blue light, our Cu_29_ was tested as a photothermal agent, and a photothermal efficiency of 33% was obtained. Finally, we showed that Cu_29_ can work as an effective catalyst for the azide–alkyne click chemistry reaction, with 91% product yield in just 1 h of reaction, and it remains consistent with various alkynes. Moving forward, we hope that our work will provide important information to researchers about the synthetic design of high-yield procedures to prepare Cu NCs and provide details about the ambidexterity of the Cu_29_ NCs in photothermal conversion and catalysis.

## Experimental Section

### Synthesis of Cu_29_(CHT)_18_(TPP)_4_H_10_·BF_4_

In a 10 mL round-bottom flask, 105 mg (0.33 mmol) of tetrakis­(acetonitrile)­copper­(I) tetrafluoroborate [(Cu­(CH_3_CN)_4_·BF_4_] was dissolved in a mixture of 6 mL of a 1:1 chloroform:acetonitrile mixture. After 5 min of stirring, 57 mg (0.22 mmol) triphenylphosphine (PPh_3_) was added, followed by the addition of 27 μL (0.22 mmol) cyclohexanethiol (C_6_H_11_SH). The reaction mixture was stirred for 10 min to ensure that all of the precursors were fully dissolved. Next, 50 mg (0.55 mmol) borane *tert*-butylamine complex [(CH_3_)_3_CNH_2_·BH_3_] (dissolved in 2 mL methanol) was quickly added to the reaction mixture. Over time, the clear solution slowly turned pale yellow and finally dark red. The reaction was allowed to run for 4 h, after which the solvent was removed via rotary evaporation. The NCs were washed with acetonitrile 3 times to remove the precursors, and crystallization was conducted by any of the following procedures: (i) the washed NCs were dissolved in 1 mL of chloroform and subjected to vapor diffusion of *n*-hexane for 1 week at 4 °C, or (ii) the washed NCs were dissolved in a 2:1:1 mixture of chloroform:toluene:hexane and kept for slow evaporation at room temperature for 3-4 days. Rhombohedral red–orange crystals of Cu_29_ were obtained, which were collected for further experiments. The yield was about 71% (Cu atom basis).

### Synthesis of Cu_29_(CHT)_18_(TPP)_4_D_10_·BF_4_

The synthetic procedure is similar to that above, except that the reducing agent (borane *tert*-butylamine complex, (CH_3_)_3_CNH_2_·BH_3_) was replaced by its deuterated analogue, (CH_3_)_3_CNH_2_·BD_3_.

## Supplementary Material



## Notes

Details of the Cu_29_ characterization, X-ray crystallography, photothermal and catalytic evaluation, and supporting Figures S1–S19 and Tables S1–S6 (PDF)
