# Supplementary material for: High-Yield Synthesis of Cu29 Nanoclusters and Their Applications in Photothermal Conversion and Catalysis
Source: Inorg Chem. 2025 Aug 22;64(35):17687–95. doi: 10.1021/acs.inorgchem.5c01411 (PMC12421667; doi:10.1021/acs.inorgchem.5c01411)
Supplement: Supplementary file 1 [file ic5c01411_si_001.pdf]

Supporting Information:

High-Yield Synthesis of Cu_{29} Nanocluster and Its Applications in Photothermal Conversion and Catalysis

Avirup Sardar,[a] Yitong Wang,[a] Abhrojyoti Mazumder,[a] Guiying He,[a] Christopher G. Gianopoulos,[b] Kristin Kirschbaum,[b] and Rongchao Jin[a]*

[a] Department of Chemistry, Carnegie Mellon University, Pittsburgh, Pennsylvania 15213, United States
[b] Department of Chemistry and Biochemistry, University of Toledo, Toledo, Ohio 43606, United States
Corresponding author: (R.J.) rongchao@andrew.cmu.edu

S1. Experimental

S1.1. Chemicals

Tetrakis(acetonitrile)copper(I) tetrafluoroborate ($Cu(CH_3CN)_4BF_4$, 97%, Aldrich), triphenylphosphine (≥99%, Aldrich), cyclohexanethiol ($C_6H_{11}SH$, 98%, Aldrich), tert-Butylamine borane ($(CH_3)_3CNH_2{\cdot}BH_3$, powder, 98%, Aldrich), sodium borodeuteride ($NaBD_4$, 98 atom % D, Aldrich). All the chemicals used in the catalysis experiments were purchased from Aldrich. Solvents: Acetonitrile, chloroform (HPLC grade, ≥99%, Aldrich), methanol (HPLC grade, ≥99%, Aldrich), toluene (HPLC grade, ≥99%, Aldrich), hexane (HPLC grade, ≥99%, Aldrich), dichloromethane (HPLC grade, ≥99%, Aldrich). (HPLC grade, ≥99%, Aldrich) chloroform-d (100%, 99.96 atom % D, contains 0.03% (v/v) TMS), All chemicals were used as received without further purification.

S1.2. Additional Synthesis Procedure

Synthesis of ^{t}BuNH$_2{\cdot}$BD$_3$: The deuterated tert-butylamine borane complex (^{t}BuNH$_2{\cdot}$BD$_3$) were synthesized using a previously reported method.[1,2]

S1.3. Characterization

UV-vis-NIR: The UV–vis–NIR spectra for all the Cu nanoclusters were obtained using a UV-3600 Plus spectrophotometer (Shimadzu) with a wavelength range of 185-3300 nm.

Photothermal Conversion: To evaluate the photothermal conversion, chloroform solutions of Cu_{29} NCs, with different concentrations were irradiated by a 488nm laser (1.75 W cm^{-2}). The temperature change was tracked by HSFTools HP96 Thermal Camera.

Electrospray ionization mass spectrometry (ESI-MS): ESI-MS were performed on a Waters QTOF mass spectrometer equipped with Z-spray source. The sample was dissolved in dichloromethane. The source temperature was kept at 70 °C. The sample was directly infused into the chamber at 160 µL/min. The spray voltage was kept at 4.65 kV and the cone voltage at 80 V.

Transient Absorption analyses: The broadband femtosecond (fs) transient absorption measurements were performed using a home-built pump-probe setup based on a commercial Yb:KGW amplified laser system (Carbide CB3, Light Conversion) with a repetition rate of 90 kHz, center wavelength of 1030 nm. The pump pulses of 400 nm were generated by an optical parametric amplifier (Orpheus-F, Light Conversion). The white continuous probe light was generated by focusing the fundamental output from Carbide laser onto an yttrium aluminum garnet (YAG) crystal. Probe pulses are modulated by an electro-optic modulator and mechanically delayed in time relative to the pump pulse using a delay stage and retroreflector before white light generation. The pump pulses induced change in the transmission of

probe pulse was collected by a miniature spectrometer and fast line-scan camera (Teledyne e2V Octoplus USB) using a modified shot-by-shot detection scheme.

S1.4. Cu_{29}-catalyzed azide–alkyne cycloaddition reactions

A 10 mL vial was charged with 0.02 mol% Cu_{29} NCs, 0.5mmol of benzyl azide and 0.55mmol any of the five acetylenes (Phenylacetylene, 3-Ethynyltoluene, 4-Ethynyltoluene or 4-tertButylphenylacetylene and 1-Octyne) under N_2 atmosphere. 3mL d-chloroform containing 0.03% (v/v) TMS was used as a solvent. The reaction mixture was then irradiated with blue LED (50W equivalent, Sunlite 81467 LED PAR20) 5 cm away from the reaction vial for 1 hr. The temperature was maintained around 25°C. All reactions were conducted under a N_2 atmosphere in thoroughly cleaned and oven-dried glassware. After 1hr column chromatography was conducted to separate the products and a a Büchi rotary evaporator was used to concentrate the products. All the yields are reported for the isolated products.

Isolated compounds were characterized by ^{1}H NMR using a 500MHz Bruker NMR spectrometer at 298K. Position of the signals were recorded in δ ppm and measured from the center of the said signal, multiplets were provided as a range. Splitting patterns are reported as singlet (s), doublet (d), triplet (t), quartet (q), quintet (quin), septet (sept), and multiplet (m), or a combination of them. The TMS peak at 0 ppm was used as a reference for all the spectra. Coupling constants (J) are reported in Hz.

S1.5. X-ray Crystallography

A small crystal, approximate dimensions 0.010 mm x 0.020 mm x 0.045 mm, was used for the X-ray crystallographic analysis. The X-ray intensity data were measured using Cu-radiation, λ = 1.54178 Å. All crystals analyzed showed poor quality, partially due to rapid decomposition and macroscopic twinning. The final crystal selected was integrated as a three-component twin. However, final refinement with an HKLF4 file based on the largest component gave the best model.

The frames were integrated with the Bruker SAINT software package using a narrow-frame algorithm. The integration of the data using a trigonal unit cell yielded a total of 65,357 reflections to a maximum θ angle of 48.24° (1.03 Å resolution), of which 6231 were independent (average redundancy 10.489, completeness = 87.1%, R_{int} = 13.15%, R_{sig} = 12.51%) and 3,733 (59.91%) were greater than $2\sigma(F^2)$. The final cell constants were later converted to the standard hexagonal setting: a = 21.155(2) Å, b = 21.155(2) Å, c = 86.5621(10) Å, volume = 33,549.(8) Å^3. Data were corrected for absorption effects using the Multi-Scan method (TWINABS). The ratio of minimum to maximum apparent transmission was 0.674. The calculated minimum and maximum transmission coefficients (based on crystal size) are 0.3231 and 0.4452.

The structure was solved and refined with the Bruker SHELXTL Software Package, using the rhombohedral setting of the space group R -3, with Z = 2 for the formula unit, $C_{162}H_{225}Cu_{29}P_4S_{18}$. Final refinements were performed after unit cell, coordinates and hkl were transformed to the standard hexagonal setting. The final anisotropic full-matrix least-squares refinement on F^2 with 346 variables converged at R1 = 17.90%, for the observed data and wR2 = 48.11% for all data. The goodness-of-fit was 1.958. The largest peak in the final difference electron density synthesis was 1.077 $e^-/Å^3$ and the largest hole was -0.747 $e^-/Å^3$ with an RMS deviation of 0.191 $e^-/Å^3$. On the basis of the final model, the calculated density was 1.400 g/cm^3 and F(000), 14,316 e^-.

Data were reduced as a 3-component twin, the final refinement was refined against HKLF4 data which provided the best model. The data quality and crystal quality are low, despite have screened numerous (unstable) crystals. Modeling of cyclohexyl rings was challenging. Disorder models were pursued although no suitable disorder model could be obtained. For this reason, the cyclohexyl rings S3-C31-C36 and S6-C61-C66 were modeled with half occupancy. Additional conformations could not be identified with any confidence from the difference map. SIMU restraints was applied to all neighboring carbon atoms. 1,2- and 1,3- distance restraints were applied to the cyclohexyl thiolate groups (SAME S1-C16). The PPh_3 phenyl groups were constrained to the corners of a regular hexagon (AFIX 66). A BF4- anion was suggested on the

basis of MS, although it could not be located in the difference map. SQUEEZE was applied.[3] SQUEEZE suggested positions with significant electron density with a total of ca. 2000 electrons/cell. The total void volume was determined to be ~20%.

S1.6. Calculation of Photothermal Conversion Efficiency (η)

The photothermal conversion efficiency was caluculated by reported methods.[4–8]
The photothermal conversion efficiency (η) equation is as follows:

$$\eta = \frac{\mathrm{hS\Delta T_{max}}}{I\,(1-10^{-A_\lambda})}$$

(I =laser power density (1.75 W.cm^{-2}), A_λ = absorbance of NCs at the irradiated wavelength, h = heat transfer coefficient, S = total surface area of the vial, ΔT_{max} = maximum temperature change)

To find out the hS, we replace it with the the heat time constant, τ_s . The relation between hS and τ_s is given by:

$$\tau_s = \frac{\sum m_i C_{p,i}}{hS}$$

(m_i and $C_{p,i}$ are the mass and heating capacity of the systems respectively. The mass of the NCs were too negligible in compared to the NCs, so the values for the solvent chloroform is taken. For chloroform: m_i = 0.74 g, $C_{p,i}$= 0.96 J/(g °C))

During the cooling time (when the laser is off), we can find out the τ_s by introducing a dimensionless constant θ, which is basically:

$$\theta = \frac{\mathrm{T} - T_{surr}}{T_{max} - T_{surr}}$$

(T = temperature of system, T_{max} = maximum temperature reached, T_{surr} = initial temperature)

$$t = -\tau_s ln\theta$$

$\tau_s$is calculated from the slope of cooling time (t) *vs* –ln θ plot, and plugging in the values in the position of hS.

$\tau_s$is calculated from the slope of the plot of cooling time t *vs* –ln θ, and plugging in the values, we can obtain τ_s = 154.9 and then hS. Finally, η of Cu_{29} is calculated to be 33%.

(note: ΔT_{max} = 22.4°C, I = 1.75 W·cm^{-2} and A_λ = 0.4 OD)

S2. Supporting Figures:

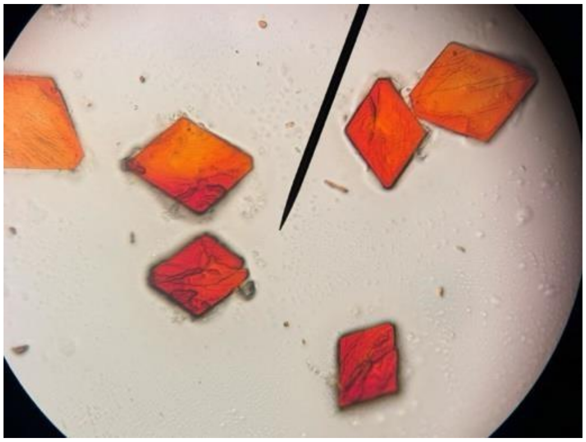

Figure S1. Optical microscopic image of Cu_{29} crystals.

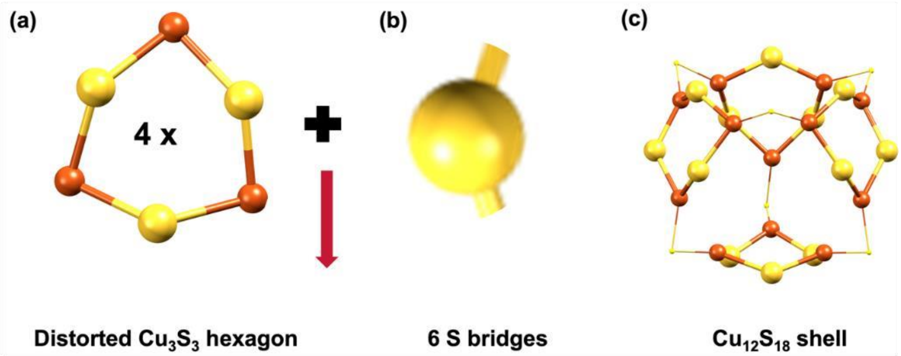

Figure S2. Shell structure of the Cu_{29} NCs a) 4 distorted Cu_3S_3 hexagons, b) 6 S^- bridges, and c) overall $Cu_{12}S_{18}$ shell

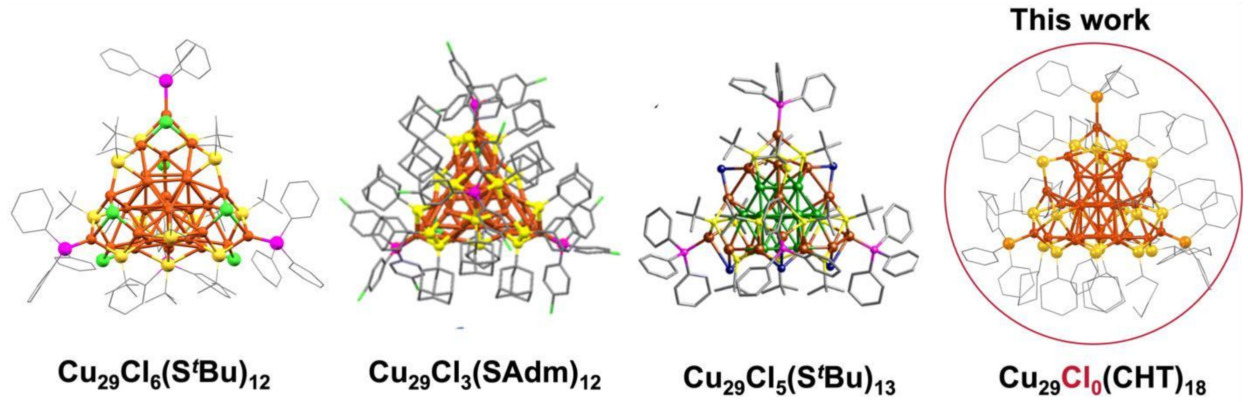

Figure S3. Comparison between the reported Cu_{29}-CHT-TPP clusters[9–11] and this work (note: our NC having no Cl (green) in the crystal structure).

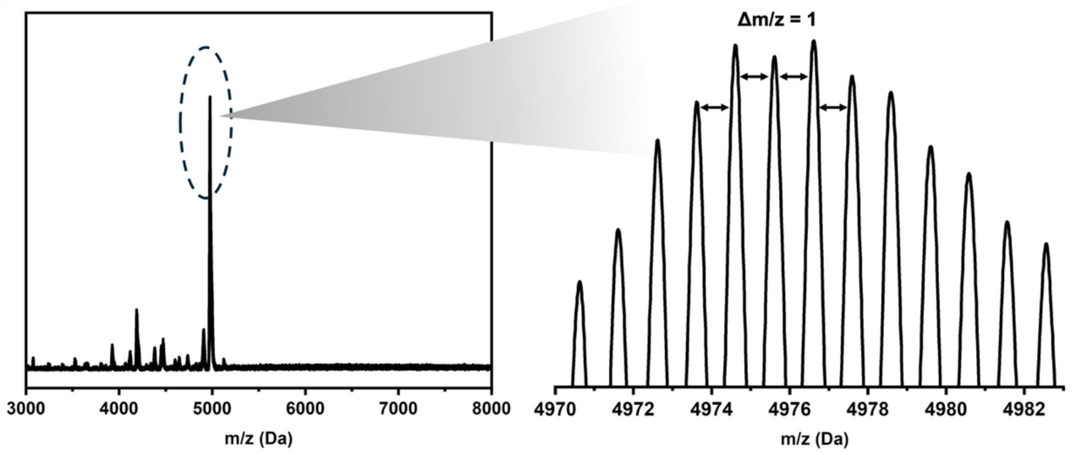

Figure S4. Magnified image of the peak at m/z 4976.56 in positive mode ESI-MS spectrum. The Δm/z = 1 proves that the cluster is in +1 charge state.

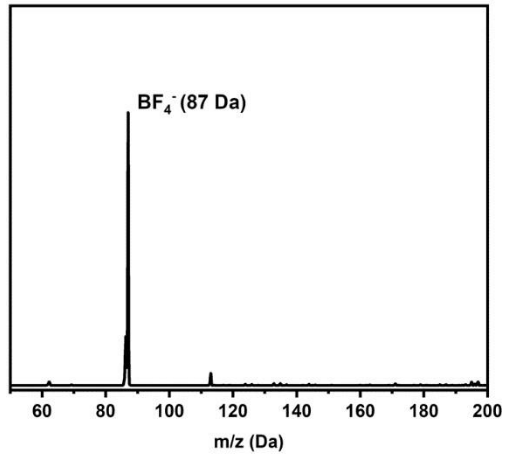

Figure S5. Negative mode ESI-MS for the Cu_{29} NCs, the peak at 87 Da corresponds to the BF_4^- counter anion.

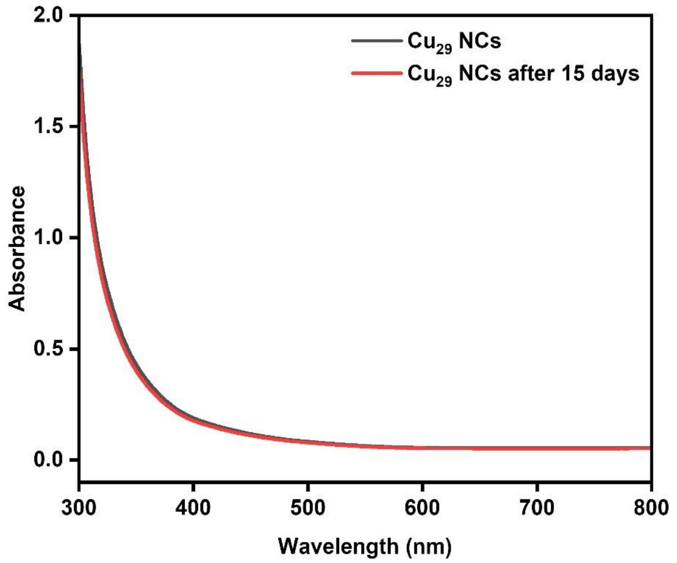

Figure S6. Photostability test of the Cu_{29} NC evaluated by UV-vis spectra before and after 15 days of storage.

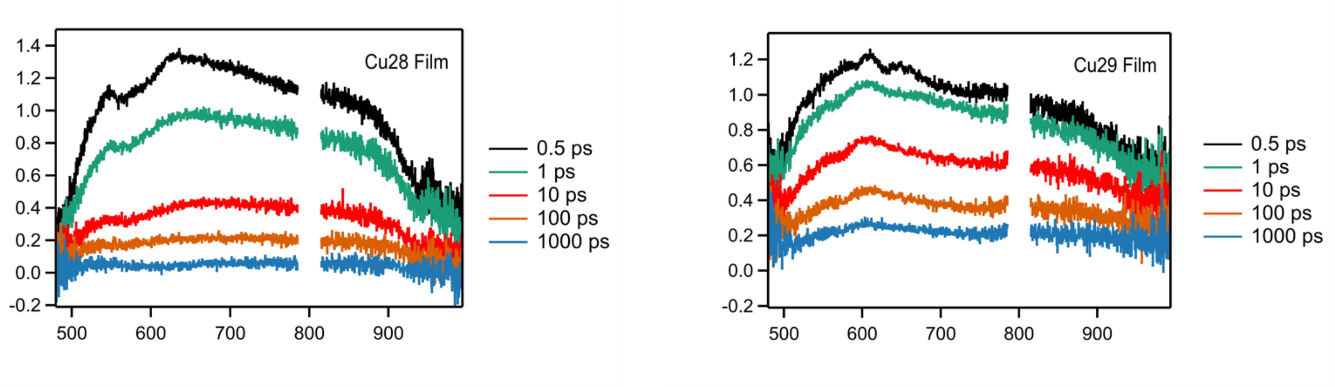

Figure S7. Transient absorption spectra of the Cu_{29} and Cu_{28} films at different delay times.

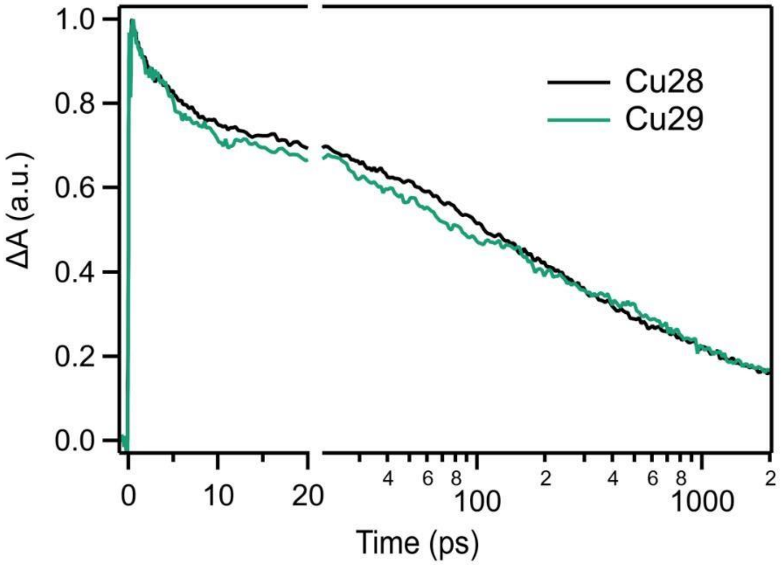

Figure S8. Tri-exponential fitting for the Cu_{29} and Cu_{28} NCs. The fitted time-constants:

	t1	t2	t3
Cu_{28}	5.9 ps (24%)	137 ps (40%)	2. 2 ns (36%)
Cu_{29}	6.3 ps (25%)	91 ps (33%)	1.8 ns (42%)

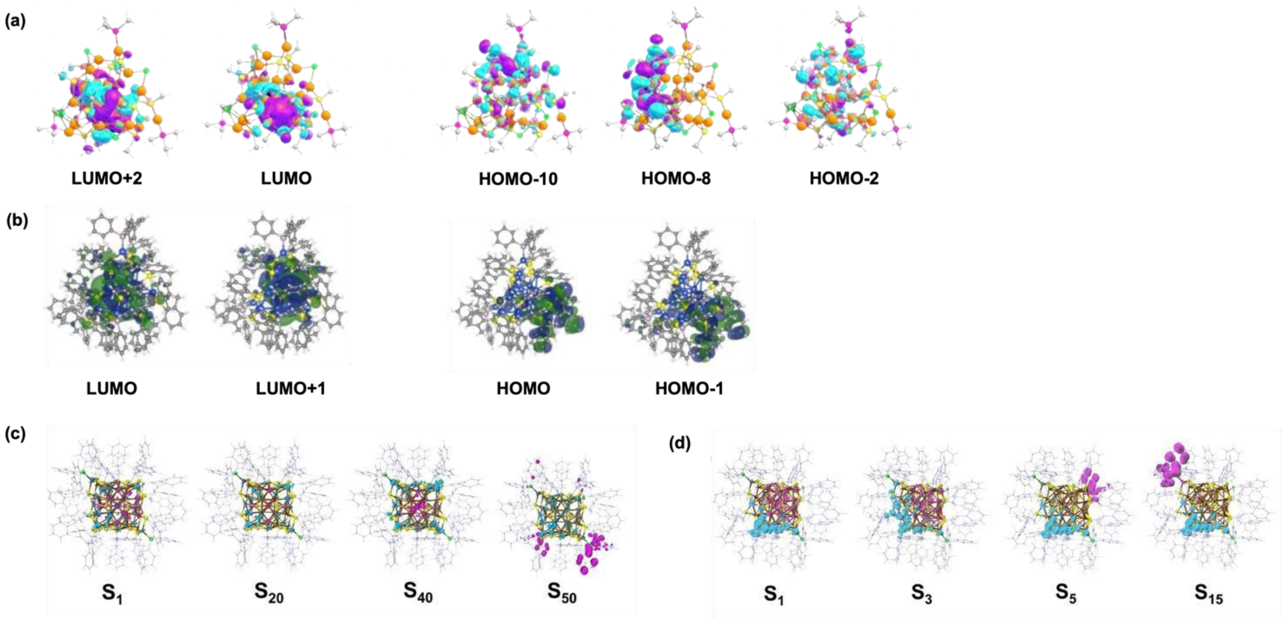

Figure S9. Frontier molecular orbitals of (a) reported optimized Cu_{29} NC, (b) optimized Cu_{28} NC and hole-electron maps for four representative excited states for the optimized (c) Cu_{58} and (d) Cu_{57}. Reproduced with permission from ref.[16-18]. © Wiley-VCH GmbH

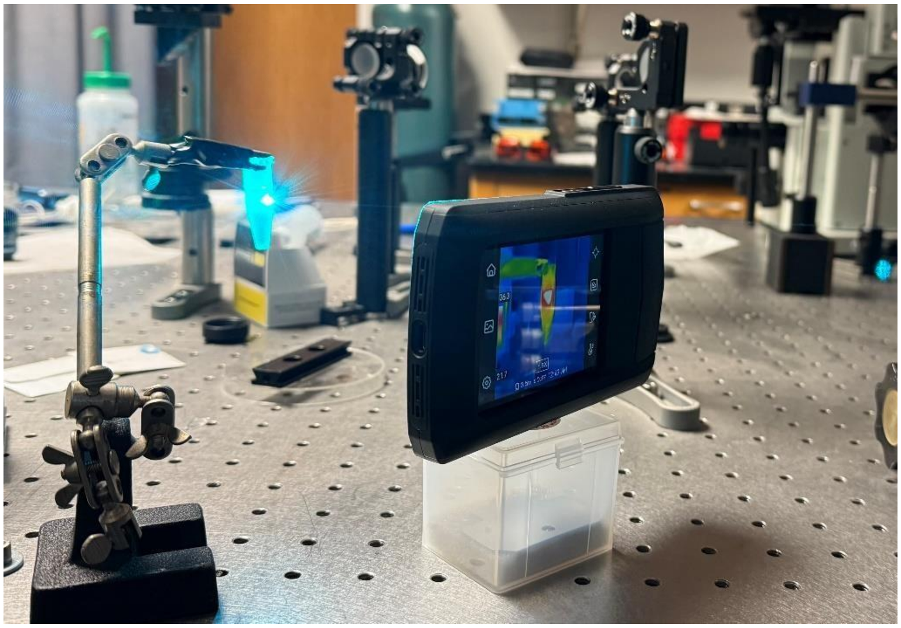

Figure S10. Photothermal conversion experimental setup (a 488 nm laser, 1.75 Wcm^{-2}, a thermal camera, and the NCs dissolved in solution (0.4 OD at 488 nm).

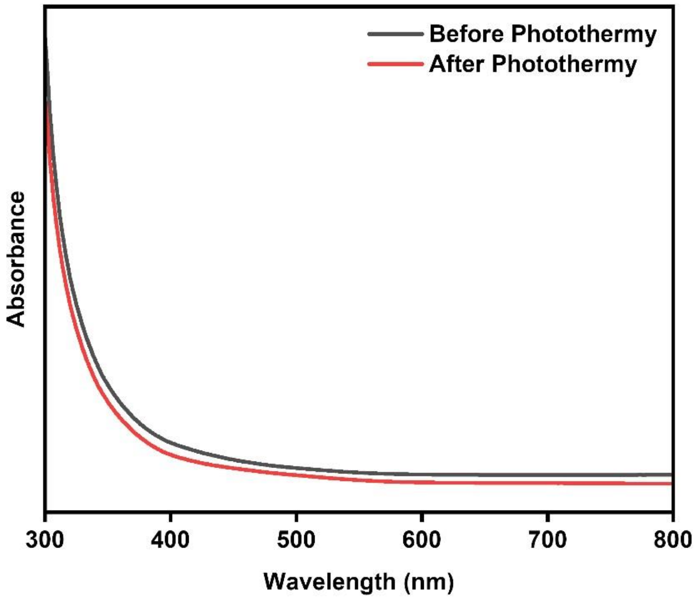

Figure S11. UV-vis spectra of the Cu_{29} NCs before and after photothermal conversion experiments.

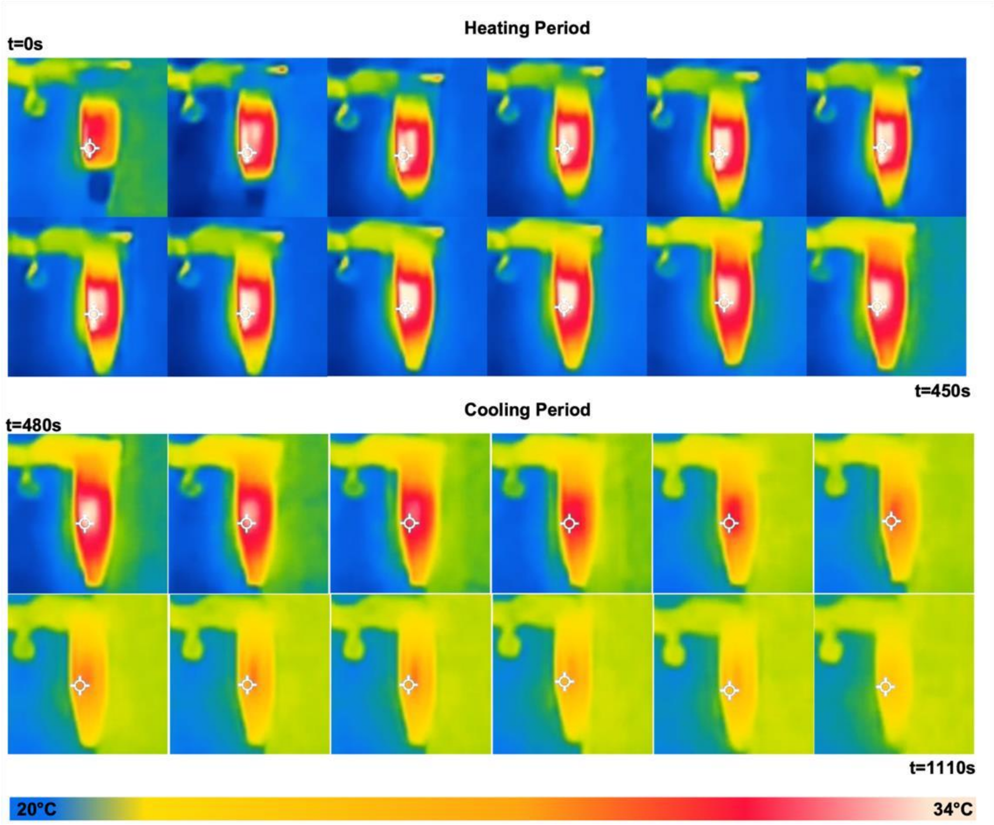

Figure S12. Thermal camera images of heating and cooling cycles of NCs at 0.2 OD.

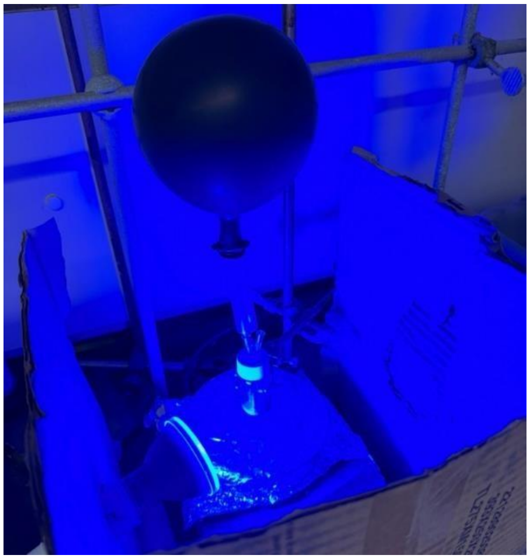

Figure S13. Setup for the blue-LED irradiated catalysis experiments.

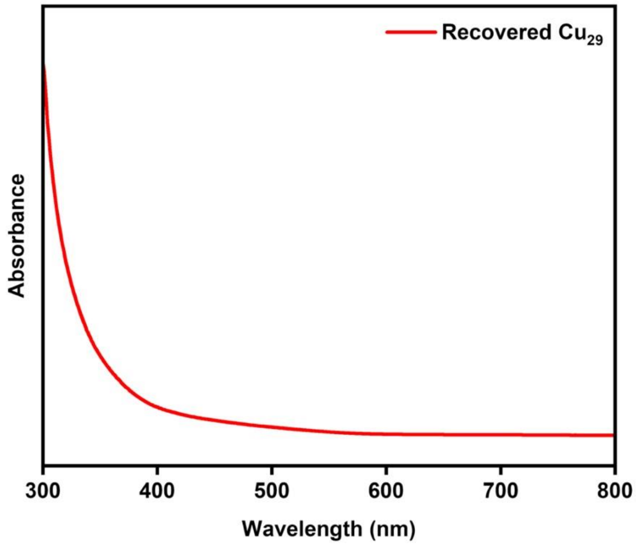

Figure S14. UV-vis spectra of the recovered Cu_{29} NCs after catalysis experiments.

1-benzyl-4-phenyl-1H-1,2,3- triazole:

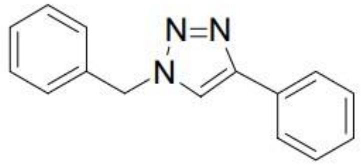

1H NMR (500 MHz, $CDCl_3$): δ 7.38 (d, 2H), 7.66 (s, 1H), 7.40–7.25 (m, 8H), 5.56 (s, 2H).[12]

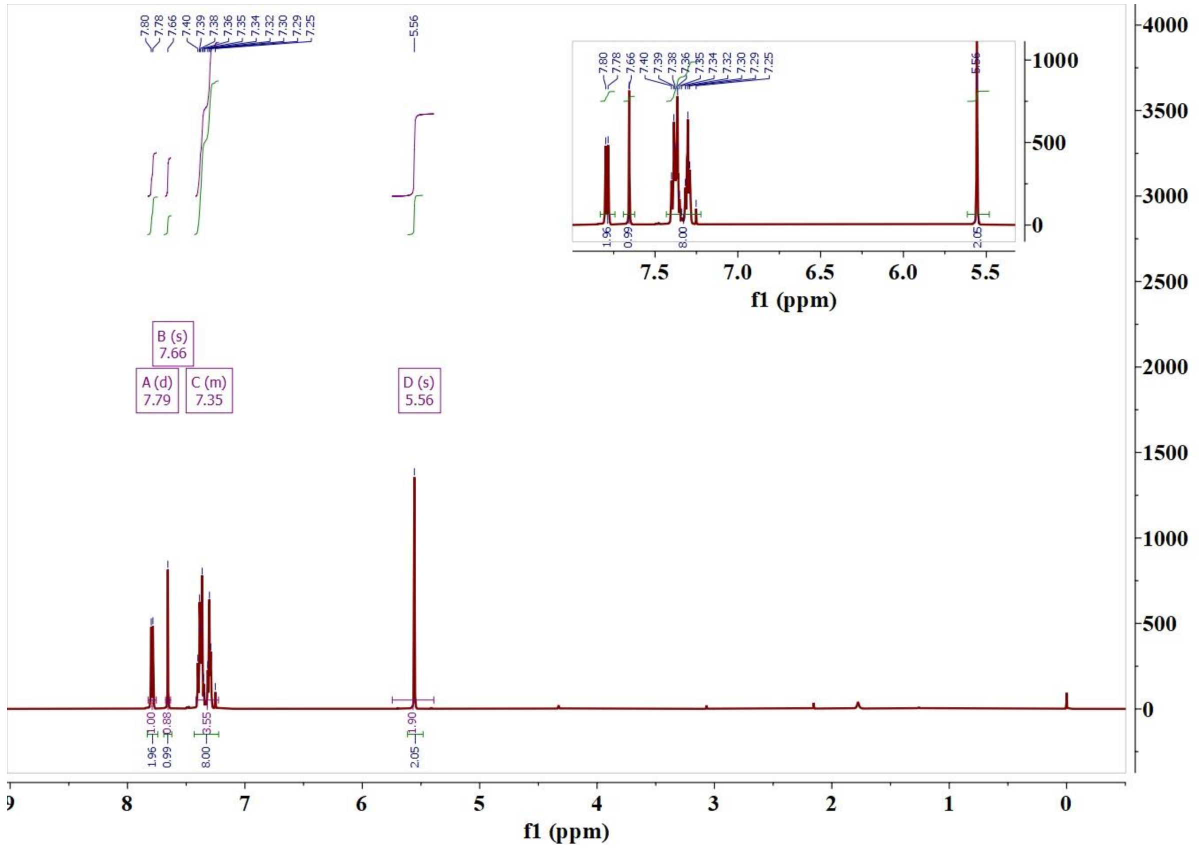

Figure S15. NMR spectrum of 1-benzyl-4-phenyl-1H-1,2,3- triazole. **1-Benzyl-4-(4-methylphenyl)-1H-1,2,3-triazole:**

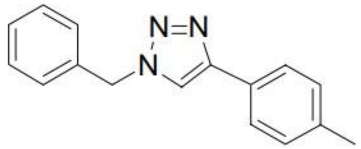

1H NMR (500 MHz, $CDCl_3$): δ 7.71 (d, J = 8.4 Hz, 2H), 7.62 (s, 1H), 7.44 – 7.29 (m, 5H), 7.22 (d, J=7.7 Hz, 2H), 5.57 (s, 2H), 2.37 (s, 3H).[13]

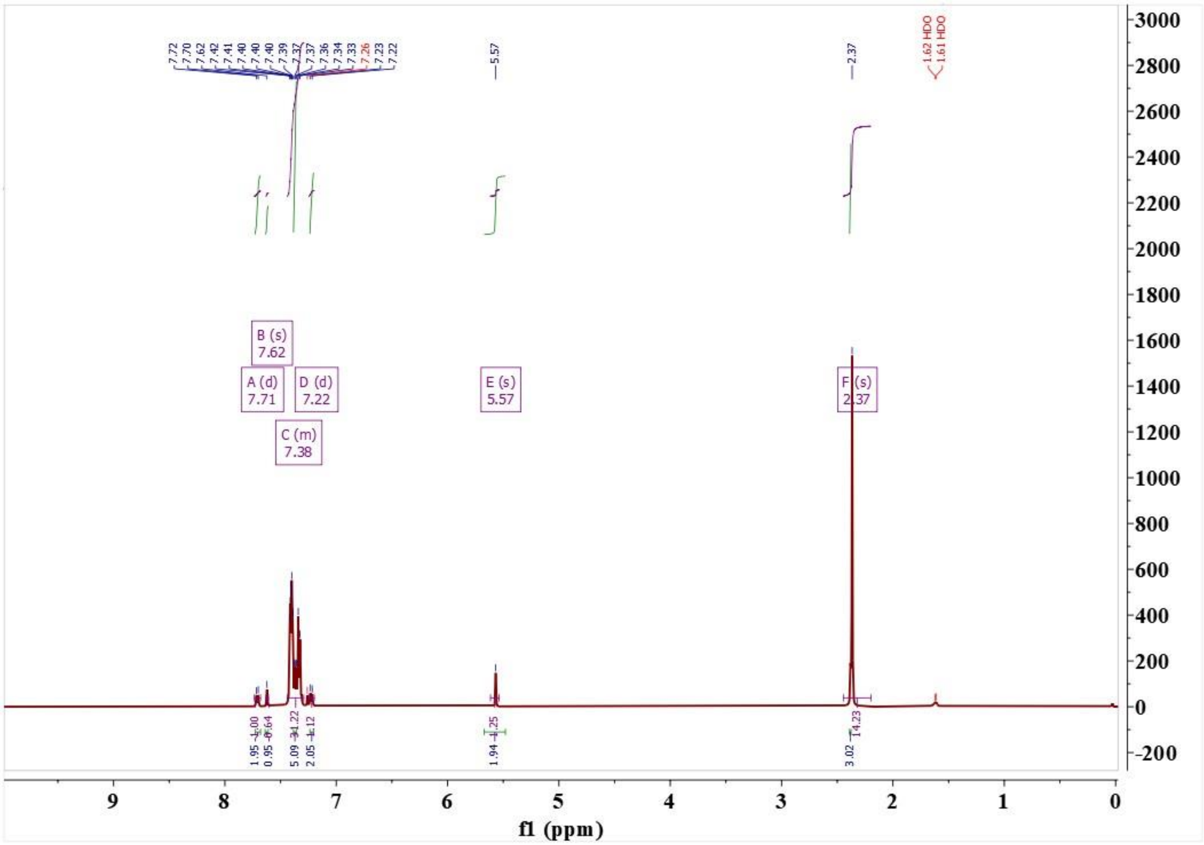

Figure S16. NMR spectrum of 1-Benzyl-4-(4-methylphenyl)-1H-1,2,3-triazole**. 1-Benzyl-4-(3-methylphenyl)-1H-1,2,3-triazole**:

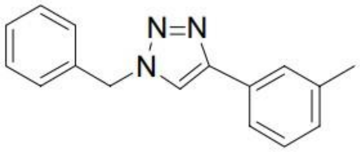

1H NMR (500 MHz, $CDCl_3$): δ 7.68 – 7.52 (m, 3H), 7.42 – 7.10 (m, 7H), 5.56 (s, 2H), 2.32 (s, 3H)[13]

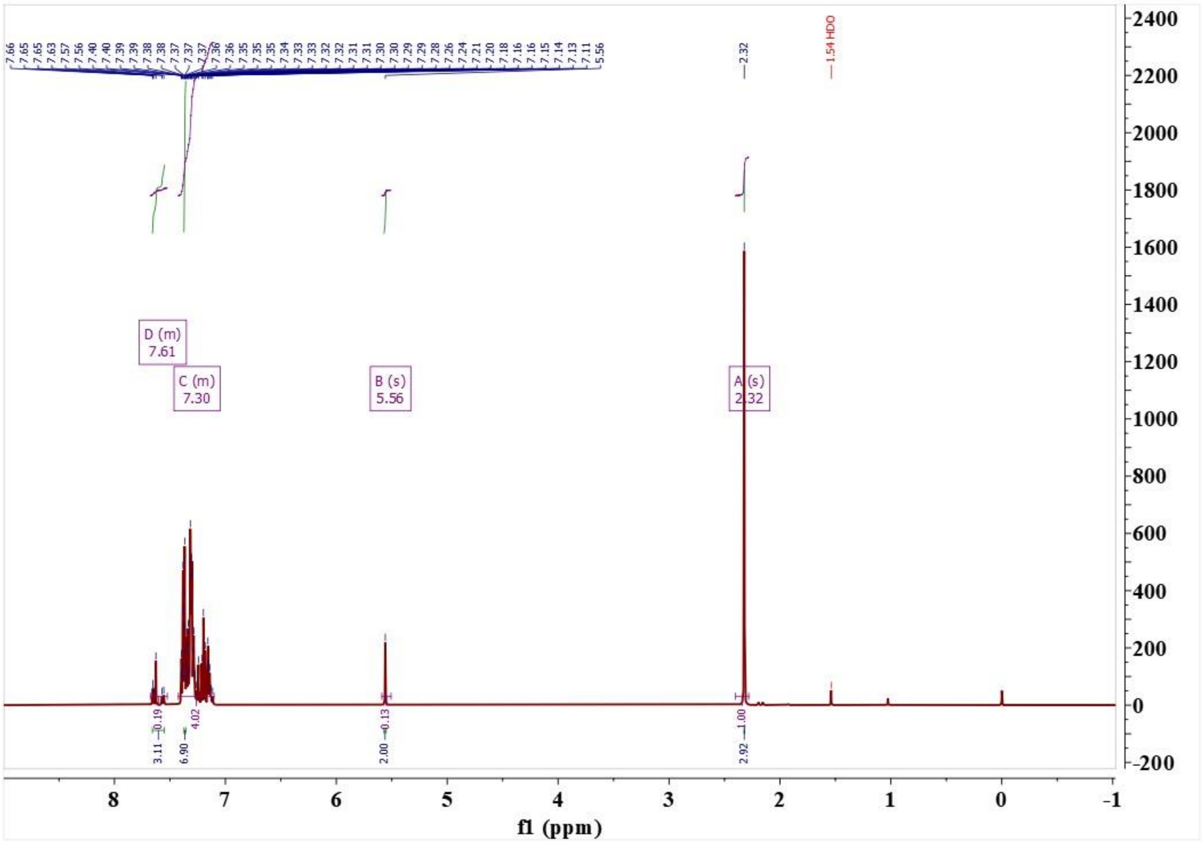

Figure S17. NMR spectrum of 1-Benzyl-4-(3-methylphenyl)-1H-1,2,3-triazole. **1-benzyl-4-(4-(tert-butyl)phenyl)-1H-1,2,3-triazole:**

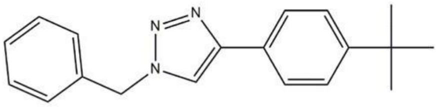

^{1}H NMR (500 MHz, $CDCl_3$): δ 7.72 (d, *J* = 8.4 Hz, 2H), 7.63 (s, 1H), 7.43 – 7.27 (m, 7H), 5.54 (s, 2H), 1.32 (s, 9H).[14]

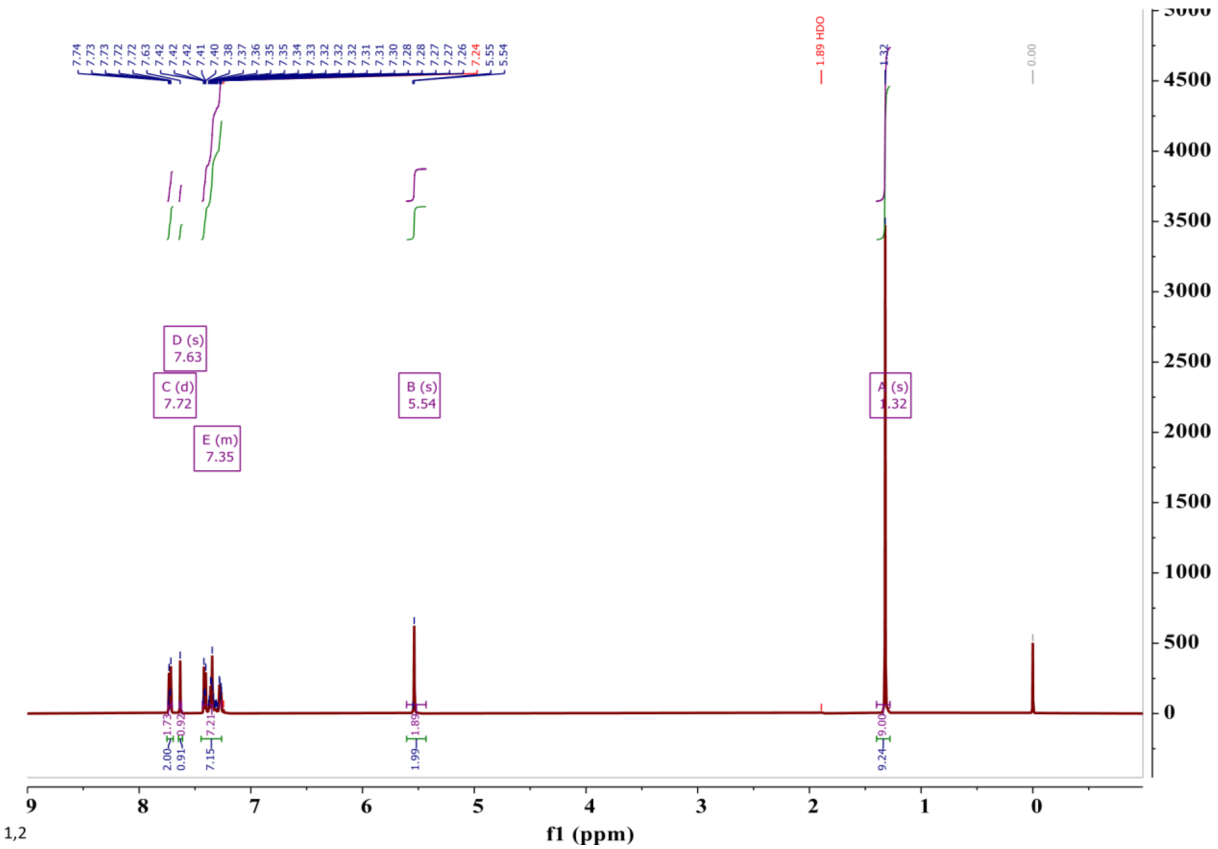

Figure S18. NMR spectrum of 1-benzyl-4-(4-(tert-butyl)phenyl)-1H-1,2,3-triazole. **1-benzyl-4-hexyl-1H-1,2,3-triazole**:

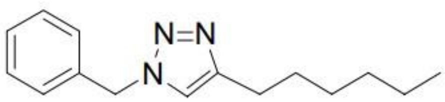

^{1}H NMR (500 MHz, $CDCl_3$): δ 0.86 (t, 3H), 1.25-1.33 (m, 6H), 1.58-1.66 (m, 2H), 2.66 (t, 3 J(H,H) = 7.56 Hz, 2H), 5.50 (s, 2H), 7.18–7.41 (m, 6H)[15]

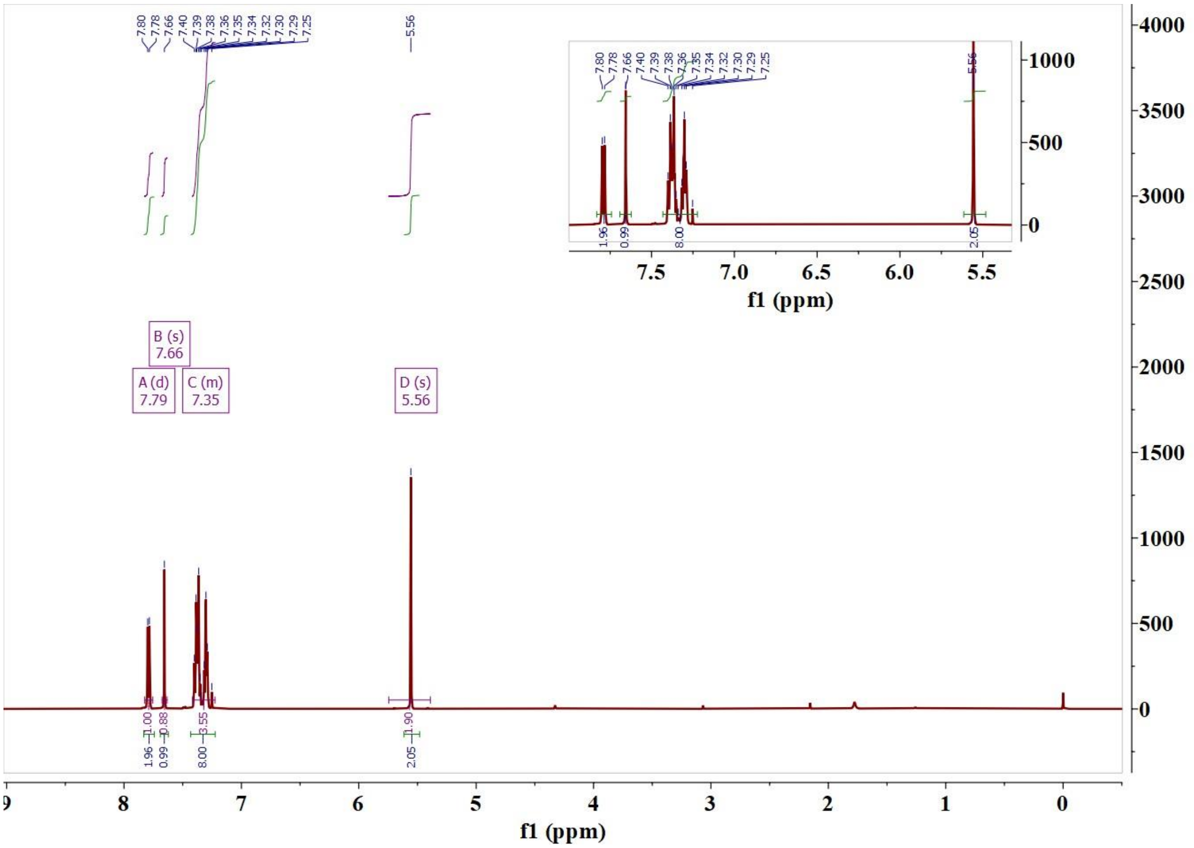

Figure S19. NMR spectrum of 1-benzyl-4-hexyl-1H-1,2,3-triazole.

S3. Supporting Tables:

Table S1. Yields of Cu_{29} under various reaction conditions

Reaction Conditions			Cu_{29} Yield (%)
Cu salt:TPP ratio	Solvent	Reducing agent	
1:1	$CHCl_3$:CH_3CN (1:1)	$^tBuNH_2 \cdot BH_3$	Mainly Cu_{14}
**1.5:1**	**$CHCl_3$:CH_3CN (1:1)**	**$^tBuNH_2 \cdot BH_3$**	**71%**
2:1	$CHCl_3$:CH_3CN (1:1)	$^tBuNH_2 \cdot BH_3$	~20%
1.5:1	$CHCl_3$:CH_3CN (1:1)	$NaBH_4$	~30%
1.5:1	$CHCl_3$	$^tBuNH_2 \cdot BH_3$	~30%
1.5:1	CH_3CN	$^tBuNH_2 \cdot BH_3$	0%

Table S2. Sample and crystal data for Cu_{29}

**Chemical formula**	$C_{162}H_{225}Cu_{29}P_4S_{18}$	
**Formula weight**	4716.03 g/mol	
**Temperature**	250(2) K	
**Wavelength**	1.54178 Å	
**Crystal size**	0.010 x 0.020 x 0.045 mm	
**Crystal system**	trigonal	
**Space group**	R -3	
**Unit cell dimensions**	a = 21.155(2) Å	α = 90°
	b = 21.155(2) Å	β = 90°
	c = 86.5621(10) Å	γ = 120°
**Volume**	33549.0 (8) $Å^3$	
**Z**	6	
**Density (calculated)**	1.400 g/cm^3	
**Absorption coefficient**	4.977 mm^{-1}	
**F(000)**	14,316	

Table S3. Data collection and structure refinement for Cu_{29}

**Theta range for data collection**	2.46 to 48.24°	
**Reflections collected**	65357	
**Independent reflections**	6231 [R(int) = 0.1315]	
**Coverage of independent reflections**	87.1%	
**Absorption correction**	Multi-Scan	
**Max. and min. transmission**	0.4452 and 0.3231	
**Structure solution technique**	Intrinsic phasing	
**Structure solution program**	XT, VERSION 2018/2	
**Refinement method**	Full-matrix least-squares on F^2	
**Refinement program**	SHELXL-2019/2 (Sheldrick, 2019)	
**Function minimized**	$\Sigma\ w(F_o^2 - F_c^2)^2$	
**Data / restraints / parameters**	6231 / 285 / 346	
**Goodness-of-fit on F^2**	1.958	
**Final R indices**	3733 data; I>2σ(I)	R1 = 0.1790, wR2 = 0.4678
	all data	R1 = 0.2357, wR2 = 0.4811
**Weighting scheme**	$w=1/[\sigma^2(F_o^2)+(0.1600P)^2]$ where $P=(F_o^2+2F_c^2)/3$	
**Largest diff. peak and hole**	1.077 and -0.747 eÅ^{-3}	
**R.M.S. deviation from mean**	0.191 eÅ^{-3}	

Table S4. Atomic coordinates and equivalent isotropic atomic displacement parameters (Å^2) for Cu_{29}

U(eq) is defined as one third of the trace of the orthogonalized U_{ij} tensor.

	x/a	y/b	z/c	U(eq)
Cu1	0.8759(2)	0.8719(2)	0.75044(6)	0.0983(17)
Cu2	0.8358(3)	0.8184(3)	0.72231(6)	0.113(2)
Cu3	0.9783(3)	0.8142(3)	0.72289(6)	0.116(2)
Cu4	0.0360(3)	0.9590(3)	0.72400(6)	0.0931(17)
Cu5	0.9899(3)	0.8679(3)	0.75076(5)	0.0951(17)
Cu6	0.000000	0.000000	0.74973(9)	0.087(3)
Cu7	0.9538(3)	0.9108(2)	0.77378(6)	0.0990(18)
Cu8	0.8660(3)	0.9355(3)	0.79128(7)	0.124(2)
Cu9	0.7704(3)	0.8882(3)	0.76215(6)	0.121(2)
Cu10	0.1375(3)	0.8466(3)	0.72649(6)	0.124(2)
Cu11	0.000000	0.000000	0.81589(11)	0.144(4)
P1	0.000000	0.000000	0.84147(18)	0.102(5)
P2	0.1925(6)	0.7894(6)	0.71692(12)	0.121(4)
C1P	0.9305(16)	0.0098(17)	0.8509(4)	0.186(14)

	x/a	y/b	z/c	U(eq)
C2P	0.868(2)	0.9821(16)	0.8420(3)	0.188(14)
C3P	0.8088(16)	0.9887(16)	0.8469(4)	0.209(15)
C4P	0.8127(15)	0.0229(17)	0.8609(4)	0.194(14)
C5P	0.875(2)	0.0506(14)	0.8699(3)	0.172(13)
C6P	0.9343(15)	0.0440(15)	0.8649(4)	0.160(13)
C7P	0.129(2)	0.6894(16)	0.7090(4)	0.213(16)
C8P	0.056(3)	0.669(2)	0.7069(4)	0.224(16)
C9P	0.0052(16)	0.598(3)	0.7030(4)	0.251(17)
C10P	0.027(3)	0.5465(16)	0.7011(4)	0.263(17)
C11P	0.100(3)	0.567(2)	0.7031(4)	0.242(17)
C12P	0.1509(17)	0.638(3)	0.7071(4)	0.229(16)
C13P	0.2465(16)	0.827(2)	0.7022(3)	0.194(14)
C14P	0.2434(15)	0.7887(13)	0.6889(5)	0.179(13)
C15P	0.2817(18)	0.826(2)	0.6758(3)	0.175(13)
C16P	0.3232(15)	0.902(2)	0.6760(3)	0.180(13)
C17P	0.3263(16)	0.9402(13)	0.6893(5)	0.202(14)
C18P	0.2880(19)	0.903(2)	0.7024(3)	0.198(14)
C19P	0.2411(15)	0.7737(13)	0.7315(3)	0.115(10)
C20P	0.3088(17)	0.7829(14)	0.7275(3)	0.170(13)
C21P	0.3531(11)	0.7782(14)	0.7388(4)	0.181(13)
C22P	0.3296(15)	0.7644(14)	0.7541(3)	0.160(12)
C23P	0.2619(16)	0.7553(13)	0.7581(2)	0.153(12)
C24P	0.2176(11)	0.7599(12)	0.7468(3)	0.118(10)
S1	0.0872(4)	0.9043(4)	0.71155(9)	0.081(3)
C11	0.0751(17)	0.9028(16)	0.6903(3)	0.122(11)
C12	0.094(2)	0.8481(18)	0.6829(3)	0.150(12)
C13	0.090(2)	0.8476(19)	0.6645(4)	0.165(13)
C14	0.119(2)	0.924(2)	0.6575(4)	0.166(13)
C15	0.090(2)	0.970(2)	0.6645(4)	0.183(13)
C16	0.1031(19)	0.9735(17)	0.6845(3)	0.145(12)
S2	0.0364(5)	0.7959(4)	0.74418(10)	0.090(3)
C21	0.9857(18)	0.7025(15)	0.7524(5)	0.174(13)
C22	0.0183(19)	0.6562(17)	0.7467(5)	0.178(14)
C23	0.974(2)	0.5747(19)	0.7534(5)	0.211(15)
C24	0.891(2)	0.5420(19)	0.7517(5)	0.216(15)
C25	0.8619(19)	0.5861(19)	0.7593(5)	0.195(14)
C26	0.9121(18)	0.6779(17)	0.7542(5)	0.184(14)
S3	0.8731(5)	0.7494(6)	0.71203(14)	0.136(4)
C31	0.828(3)	0.6624(19)	0.7012(5)	0.24(3)
C32	0.761(2)	0.654(4)	0.6926(7)	0.22(3)
C33	0.783(4)	0.697(5)	0.6765(8)	0.22(3)

	x/a	y/b	z/c	U(eq)
C34	0.855(4)	0.709(4)	0.6696(7)	0.22(3)
C35	0.874(4)	0.652(5)	0.6742(6)	0.21(3)
C36	0.876(4)	0.644(4)	0.6944(7)	0.22(3)
S4	0.7557(4)	0.8030(5)	0.74294(10)	0.095(3)
C41	0.695(3)	0.7116(18)	0.7509(5)	0.48(3)
C42	0.650(4)	0.655(2)	0.7386(5)	0.50(3)
C43	0.589(3)	0.581(3)	0.7468(8)	0.51(3)
C44	0.616(5)	0.565(3)	0.7621(8)	0.50(3)
C45	0.640(4)	0.624(3)	0.7741(6)	0.49(3)
C46	0.667(4)	0.709(3)	0.7655(6)	0.49(3)
S5	0.7493(6)	0.8665(6)	0.78704(12)	0.137(4)
C51	0.687(2)	0.895(2)	0.7950(4)	0.237(17)
C52	0.621(2)	0.871(3)	0.7842(4)	0.238(17)
C53	0.573(2)	0.905(3)	0.7906(5)	0.263(18)
C54	0.560(2)	0.895(3)	0.8081(5)	0.278(18)
C55	0.628(3)	0.923(3)	0.8177(5)	0.259(18)
C56	0.680(2)	0.885(3)	0.8111(4)	0.242(17)
S6	0.9450(4)	0.8924(4)	0.79980(10)	0.091(3)
C61	0.879(2)	0.7957(15)	0.8036(7)	0.122(17)
C62	0.898(3)	0.7448(19)	0.7951(6)	0.131(17)
C63	0.843(3)	0.6617(19)	0.8002(6)	0.123(16)
C64	0.818(3)	0.657(3)	0.8170(6)	0.130(17)
C65	0.779(3)	0.697(3)	0.8207(7)	0.140(17)
C66	0.815(2)	0.780(2)	0.8109(8)	0.151(18)

Table S5. Data table to plot Heating and cooling curves of Cu_{29} and of cooling time vs. the negative logarithm of the temperature driving force (θ) during the cooling stage

Irradiation/ Cooling Time (s)	$T_{surroundings}$	T_{system}	ΔT (°C)	θ	-ln (θ)	Cooling time (s)
0	21.1	21.1	0			
30	21.1	22	0.9			
60	21.1	24	2.9			
90	21.1	26.8	5.7			
120	21.1	29.1	8			
150	21.1	33.6	12.5			
180	21.1	36.3	15.2			
210	21.1	38.6	17.5			
240	21.1	39.9	18.8			
270	21.1	41.6	20.5			
300	21.1	42.2	21.1			
330	21.1	42.9	21.8			
360	21.1	43	21.9			
390	21.1	43.1	22			
420	21.1	43.3	22.2			
450	21.1	43.5	22.4	0	0	0
480	21.1	42.2	21.1	0.94196	0.05979	30
510	21.1	39	17.9	0.79911	0.22426	60
540	21.1	37	15.9	0.70982	0.34274	90
570	21.1	34.8	13.7	0.61161	0.49167	120
600	21.1	33.7	12.6	0.5625	0.57536	150
630	21.1	32.1	11	0.49107	0.71117	180
660	21.1	30.8	9.7	0.43304	0.83694	210
690	21.1	29.5	8.4	0.375	0.98083	240
720	21.1	27.9	6.8	0.30357	1.19214	270
750	21.1	26.2	5.1	0.22768	1.47982	300
780	21.1	25.3	4.2	0.1875	1.67398	330
810	21.1	24.6	3.5	0.15625	1.8563	360
840	21.1	24	2.9	0.12946	2.04435	390
870	21.1	23.5	2.4	0.10714	2.23359	420
900	21.1	23	1.9	0.08482	2.46721	450
930	21.1	22.7	1.6	0.07143	2.63906	480

960	21.1	22.3	1.2	0.05357	2.92674	510
990	21.1	21.9	0.8	0.03571	3.3322	540
1020	21.1	21.8	0.7	0.03125	3.46574	570
1050	21.1	21.7	0.6	0.02679	3.61989	600
1080	21.1	21.6	0.5	0.02232	3.80221	630
1110	21.1	21.5	0.4	0.01786	4.02535	660

Table S6. Catalytic Performance comparison of Cu_{29} and Cu_{28} NCs after 1 hr of reaction

Click Reaction	Triazole Yield (%)	
	with Cu_{29}	with Cu_{28}
Benzyl Azide + phenylacetylene	91	92
Benzyl Azide + 4-ethynyltoluene	81	85
Benzyl Azide + 4-tert-butyl phenylacetylene	88	85

Supporting References:

(1) Huang, R.-W.; Yin, J.; Dong, C.; Ghosh, A.; Alhilaly, M. J.; Dong, X.; Hedhili, M. N.; Abou-Hamad, E.; Alamer, B.; Nematulloev, S.; Han, Y.; Mohammed, O. F.; Bakr, O. M. [Cu81(PhS)46(tBuNH2)10(H)32]3+ Reveals the Coexistence of Large Planar Cores and Hemispherical Shells in High-Nuclearity Copper Nanoclusters. *J. Am. Chem. Soc.* **2020**, *142* (19), 8696–8705. https://doi.org/10.1021/jacs.0c00541.

(2) Ghosh, A.; Huang, R.-W.; Alamer, B.; Abou-Hamad, E.; Hedhili, M. N.; Mohammed, O. F.; Bakr, O. M. [Cu61(StBu)26S6Cl6H14]+: A Core–Shell Superatom Nanocluster with a Quasi-J36 Cu19 Core and an “18Crown-6” Metal-Sulfide-like Stabilizing Belt. *ACS Materials Lett.* **2019**, *1* (3), 297–302. https://doi.org/10.1021/acsmaterialslett.9b00122.

(3) Spek, A. L. PLATON SQUEEZE: A Tool for the Calculation of the Disordered Solvent Contribution to the Calculated Structure Factors. *Acta Cryst C* **2015**, *71* (1), 9–18. https://doi.org/10.1107/S2053229614024929.

(4) Roper, D. K.; Ahn, W.; Hoepfner, M. Microscale Heat Transfer Transduced by Surface Plasmon Resonant Gold Nanoparticles. *J. Phys. Chem. C* **2007**, *111* (9), 3636–3641. https://doi.org/10.1021/jp064341w.

(5) Kim, B.; Shin, H.; Park, T.; Lim, H.; Kim, E. NIR-Sensitive Poly(3,4-Ethylenedioxyselenophene) Derivatives for Transparent Photo-Thermo-Electric Converters. *Advanced Materials* **2013**, *25* (38), 5483–5489. https://doi.org/10.1002/adma.201301834.

(6) Fang, L.; Fan, W.; Bian, G.; Wang, R.; You, Q.; Gu, W.; Xia, N.; Liao, L.; Li, J.; Deng, H.; Yan, N.; Wu, Z. Sandwich-Kernelled AgCu Nanoclusters with Golden Ratio Geometry and Promising Photothermal Efficiency. *Angewandte Chemie International Edition* **2023**, *62* (36), e202305604. https://doi.org/10.1002/anie.202305604.

(7) Gu, W.; Zhou, Y.; Wang, W.; You, Q.; Fan, W.; Zhao, Y.; Bian, G.; Wang, R.; Fang, L.; Yan, N.; Xia, N.; Liao, L.; Wu, Z. Concomitant Near-Infrared Photothermy and Photoluminescence of Rod-Shaped Au_{52} $(PET)_{32}$ and Au_{66} $(PET)_{38}$ Synthesized Concurrently. *Angew Chem Int Ed* **2024**, *63* (32), e202407518. https://doi.org/10.1002/anie.202407518.

(8) Wang, Z.; Zhu, Y.-J.; Han, B.-L.; Li, Y.-Z.; Tung, C.-H.; Sun, D. A Route to Metalloligands Consolidated Silver Nanoclusters by Grafting Thiacalix[4]Arene onto Polyoxovanadates. *Nat Commun* **2023**, *14* (1), 5295. https://doi.org/10.1038/s41467-023-41050-x.

(9) Das, A. K.; Biswas, S.; Pal, A.; Manna, S. S.; Sardar, A.; Mondal, P. K.; Sahoo, B.; Pathak, B.; Mandal, S. A Thiolated Copper-Hydride Nanocluster with Chloride Bridging as a Catalyst for Carbonylative C–N Coupling of Aryl Amines under Mild Conditions: A Combined Experimental and Theoretical Study. *Nanoscale* **2024**, *16* (7), 3583–3590. https://doi.org/10.1039/D3NR05912J.

(10) Bao, Y.; Wu, X.; Yin, B.; Kang, X.; Lin, Z.; Deng, H.; Yu, H.; Jin, S.; Chen, S.; Zhu, M. Structured CopperHydride Nanoclusters Provide Insight into the Surface-Vacancy-Defect to Non-Defect Structural Evolution. *Chem. Sci.* **2022**, *13* (48), 14357–14365. https://doi.org/10.1039/D2SC03239B.

(11) Ghosh, A.; Sagadevan, A.; Murugesan, K.; F. Nastase, S. A.; Maity, B.; Bodiuzzaman, M.; Shkurenko, A.; Nejib Hedhili, M.; Yin, J.; F. Mohammed, O.; Eddaoudi, M.; Cavallo, L.; Rueping, M.; M. Bakr, O. Multiple Neighboring Active Sites of an Atomically Precise Copper Nanocluster Catalyst for Efficient Bond-Forming Reactions. *Materials Horizons* **2024**, *11* (10), 2494–2505. https://doi.org/10.1039/D4MH00098F.

(12) Jahanshahi, R.; Akhlaghinia, B. CuII Immobilized on Guanidinated Epibromohydrin Functionalized γFe2O3@TiO2 (γ-Fe2O3@TiO2-EG-CuII): A Novel Magnetically Recyclable Heterogeneous Nanocatalyst for the Green One-Pot Synthesis of 1,4-Disubstituted 1,2,3-Triazoles through Alkyne–Azide Cycloaddition in Water. *RSC Adv.* **2016**, *6* (35), 29210–29219. https://doi.org/10.1039/C6RA05468D.

(13) Kamata, K.; Nakagawa, Y.; Yamaguchi, K.; Mizuno, N. 1,3-Dipolar Cycloaddition of Organic Azides to Alkynes by a Dicopper-Substituted Silicotungstate. *J. Am. Chem. Soc.* **2008**, *130* (46), 15304–15310. https://doi.org/10.1021/ja806249n.

(14) Xiao Siyang, H.; Ling Liu, H.; Yan Wu, X.; Nian Liu, P. Highly Efficient Click Reaction on Water Catalyzed by a Ruthenium Complex. *RSC Advances* **2015**, *5* (6), 4693–4697. https://doi.org/10.1039/C4RA12960A.

(15) Candelon, N.; Lastécouères, D.; Diallo, A. K.; Aranzaes, J. R.; Astruc, D.; Vincent, J.-M. A Highly Active and Reusable Copper(I)-Tren Catalyst for the “Click” 1,3-Dipolar Cycloaddition of Azides and Alkynes. *Chem. Commun.* **2008**, No. 6, 741–743. https://doi.org/10.1039/B716306A.

(16) Nematulloev, S.; Sagadevan, A.; Alamer, B.; Shkurenko, A.; Huang, R.; Yin, J.; Dong, C.; Yuan, P.; Yorov, K. E.; Karluk, A. A.; Mir, W. J.; Hasanov, B. E.; Nejib Hedhili, M.; Halappa, N. M.; Eddaoudi, M.; Mohammed, O. F.; Rueping, M.; Bakr, O. M. Atomically Precise Defective Copper Nanocluster Catalysts for

Highly Selective C−C Cross-Coupling Reactions. *Angew. Chem. Inter. Ed.* 2023, 62 (26), e202303572.
(17) Dong, C.; Huang, R.-W.; Sagadevan, A.; Yuan, P.; Gutiérrez-Arzaluz, L.; Ghosh, A.; Nematulloev, S.; Alamer, B.; Mohammed, O. F.; Hussain, I.; Rueping, M.; Bakr, O. M. Isostructural Nanocluster Manipulation Reveals Pivotal Role of One Surface Atom in Click Chemistry. *Angew. Chem. Int. Ed*. 2023, 62 (37), e202307140..
(18) Das, A. K.; Biswas, S.; Pal, A.; Manna, S. S.; Sardar, A.; Mondal, P. K.; Sahoo, B.; Pathak, B.; Mandal, S. A Thiolated Copper-Hydride Nanocluster with Chloride Bridging as a Catalyst for Carbonylative C–N Coupling of Aryl Amines under Mild Conditions: A Combined Experimental and Theoretical Study. *Nanoscale* 2024, 16 (7), 3583–3590.
